# Composite Heterogeneity Threshold (CHT) in CNT- and Oxide-Modified Woven Glass/Epoxy Composites Under Multi-Loading Conditions: Experimental Validation and Continuum Model Assessment

**DOI:** 10.3390/nano16070408

**Published:** 2026-03-27

**Authors:** Batuhan Çetin, Lütfiye Dahil

**Affiliations:** Department of Mechanical Engineering, Faculty of Engineering, Istanbul Aydin University, 34295 Istanbul, Turkey; batuhancetin@stu.aydin.edu.tr

**Keywords:** woven glass fiber composites, nanoparticle reinforcement, mechanical performance, low-velocity impact, finite element modeling, composite heterogeneity threshold

## Abstract

Glass fiber-reinforced epoxy composites were modified with carbon nanotubes (CNTs), Al_2_O_3_, and TiO_2_ nanoparticles to comparatively evaluate their influence on tensile, flexural, and low-velocity impact performance within an integrated experimental–numerical framework. Nanoparticles were incorporated at controlled weight fractions to identify dispersion-controlled reinforcement regimes and the onset of heterogeneity-driven mechanical transitions. Among all formulations, 0.5 wt% CNTs provided the most pronounced static mechanical enhancement, increasing tensile strength to 419.50 MPa (≈21% improvement over the reference GF laminate) and flexural strength to 230.23 MPa (≈26% increase). In contrast, impact performance exhibited a non-monotonic evolution; the highest absorbed energy (9.64 J) was observed at 2 wt% CNTs, indicating that dynamic energy dissipation mechanisms do not necessarily scale proportionally with static strength gains. Oxide-filled systems demonstrated stiffness-dominated behavior, where increasing filler content amplified elastic mismatch and progressively reduced strength despite modulus enhancement. Finite element simulations conducted in ANSYS LS-DYNA (MAT_022) reproduced global stiffness trends within the dispersion-controlled regime. Tensile strength predictions agreed within 0–9% at optimal CNT loading, whereas larger deviations (up to ~33%) emerged under bending-dominated loading in oxide-rich systems, reflecting amplified sensitivity to microstructural heterogeneity. The coupled evolution of stiffness–strength decoupling (SSDI) and FEM deviation (η) enabled identification of a Composite Heterogeneity Threshold (CHT), defined as the nanoparticle concentration beyond which stiffness enhancement no longer translates into proportional strength or toughness improvement. Beyond this threshold, dispersion-induced heterogeneity not only reduces mechanical efficiency but also marks the boundary of homogenized continuum model adequacy across static and dynamic loading conditions.

## 1. Introduction

The increasing demand for lightweight yet mechanically robust structural materials has accelerated the development of advanced fiber-reinforced polymer (FRP) composites across aerospace, automotive, marine, and defense sectors [1,2,3,4,5,6,7]. Among these, woven glass fiber–reinforced epoxy composites offer a favorable balance between cost efficiency, corrosion resistance, and high specific mechanical performance, making them suitable for load-bearing applications [8,9,10,11,12]. Nevertheless, their structural reliability is limited by the intrinsic brittleness of thermoset epoxy matrices and progressive fiber–matrix interfacial degradation under tensile, flexural, and impact loading, where microcrack initiation and localized stress amplification often govern failure evolution.

To mitigate these limitations, nanoscale reinforcements such as carbon nanotubes (CNTs), aluminum oxide (Al_2_O_3_), and titanium dioxide (TiO_2_) have been widely incorporated into polymer matrices to enhance interfacial stress transfer and suppress crack propagation [13,14,15,16]. CNTs, owing to their high aspect ratio and stiffness, improve tensile and flexural performance at low weight fractions (typically 0.1–0.5 wt%) [17,18,19,20,21]. However, excessive CNT loading promotes agglomeration, inducing stiffness heterogeneity and stress concentration effects that counteract reinforcement benefits [22,23,24,25].

In addition to CNTs and oxide nanoparticles, a wide range of nanofillers such as graphene nanoplatelets (GNPs), nanoclays, and silica (SiO_2_) have been extensively investigated to improve the mechanical performance of polymer composites. Graphene-based nanofillers have been reported to significantly enhance stiffness and crack resistance due to their high intrinsic modulus and large surface area, enabling efficient stress transfer within the matrix [26]. Nanoclay-reinforced systems improve interfacial bonding and barrier properties, contributing to enhanced toughness and environmental stability [27]. Similarly, spherical nanoparticles such as SiO_2_ enhance stiffness and crack deflection mechanisms, although their effectiveness strongly depends on dispersion quality and particle–matrix adhesion [28,29].

In fiber-reinforced epoxy composites, previous studies have shown that nanoparticle reinforcement efficiency is highly dependent on dispersion stability and interfacial interaction rather than filler type alone. CNT-modified glass fiber composites have demonstrated significant improvements in tensile and flexural properties at low weight fractions due to effective crack-bridging and load transfer mechanisms, whereas excessive CNT content leads to agglomeration and premature failure. Similarly, oxide nanoparticles such as Al_2_O_3_ and TiO_2_ have been reported to primarily enhance stiffness, while their contribution to strength and toughness remains sensitive to elastic mismatch and particle clustering.

Therefore, CNTs, Al_2_O_3_, and TiO_2_ were specifically selected in the present study to enable a direct comparison between dispersion-controlled reinforcement and stiffness-dominated behavior within the same composite architecture. This approach provides a systematic framework to investigate the transition from homogeneous reinforcement to heterogeneity-driven mechanical response, which forms the basis of the proposed Composite Heterogeneity Threshold (CHT). Numerous studies have investigated the role of nanoparticle reinforcement in glass fiber-reinforced epoxy composites, highlighting the strong dependence of mechanical performance on dispersion quality and interfacial interactions. While CNTs can significantly enhance load transfer efficiency under uniform dispersion conditions, their effectiveness is highly sensitive to agglomeration, which introduces localized stress concentrations and reduces structural integrity.

Similarly, oxide nanoparticles such as Al_2_O_3_ and TiO_2_ primarily contribute to stiffness enhancement due to their high elastic modulus; however, their reinforcing efficiency is strongly governed by dispersion uniformity and interfacial adhesion. Poor nanoparticle distribution may lead to stiffness mismatch, localized stress concentrations, and reduced mechanical efficiency. In addition to experimental findings, finite element studies have emphasized the importance of capturing dispersion-induced heterogeneity and anisotropic material behavior to accurately predict composite response.

In addition to experimental studies, finite element modeling approaches have been widely employed to predict stiffness and strength evolution in composite systems. These models emphasize the importance of accurately capturing material anisotropy, interfacial behavior, and dispersion-induced heterogeneity. However, discrepancies between experimental and numerical results are frequently reported, particularly in systems with non-uniform nanoparticle distribution.

Similarly, oxide nanoparticles such as Al_2_O_3_ and TiO_2_ primarily enhance stiffness due to their high elastic modulus and chemical stability [30,31,32,33]. Yet, increasing ceramic filler content intensifies modulus mismatch at the particle–matrix interface, accelerating microvoid formation and premature damage under bending or impact conditions [34,35,36]. Thus, stiffness gains do not necessarily translate into proportional improvements in strength or toughness.

Although numerous studies have investigated nanoparticle-reinforced polymer composites, most works focus on individual nanoparticle systems or isolated mechanical responses. Systematic comparative evaluation of different nanoparticle types within the same woven glass fiber/epoxy architecture and identical fabrication conditions remains scarce. Moreover, previous studies typically report peak mechanical properties without quantitatively identifying the reinforcement limit beyond which nanoparticle dispersion deteriorates and microstructural heterogeneity begins to dominate the mechanical response. Consequently, the transition from dispersion-controlled reinforcement to heterogeneity-driven mechanical degradation has not yet been rigorously quantified in woven glass fiber nanocomposites. In particular, a unified framework linking stiffness–strength decoupling, microstructural damage evolution, and the reliability limits of continuum-based numerical models has not been explicitly established in the current literature.

To address this gap, the present study introduces the concept of a Composite Heterogeneity Threshold (CHT), defined as the nanoparticle concentration at which increases in elastic stiffness no longer produce proportional improvements in strength or impact tolerance. Beyond this threshold, dispersion-induced heterogeneity, interfacial strain incompatibility, and local stiffness fluctuations begin to govern the mechanical response, reducing both structural efficiency and numerical predictability.

The proposed CHT framework is validated through a combined experimental–numerical investigation. Microstructural observations obtained via SEM and EDS are correlated with tensile, three-point bending, and low-velocity impact results to identify damage evolution mechanisms. In addition, orthotropic damage simulations implemented in ANSYS LS-DYNA (Ansys Inc., Canonsburg, PA, USA, version R11). are used to evaluate model–experimental deviation trends as a function of nanoparticle content. An orthotropic material is defined as a material whose mechanical properties differ along three mutually perpendicular directions, corresponding to the principal material axes.

This integrated approach enables objective identification of the CHT region and provides a mechanistic guideline for optimizing the stiffness–strength–toughness balance in nanoparticle-modified woven glass fiber composites. By introducing the Composite Heterogeneity Threshold (CHT), the present study establishes an experimentally validated framework that quantitatively links nanoparticle dispersion, stiffness–strength decoupling, and numerical model reliability in woven glass fiber nanocomposites.

## 2. Materials and Methods

### 2.1. Materials and Composite Fabrication

Woven E-glass fiber fabrics with a plain weave architecture (0°/90° orientation) and an areal density of 200 g/m^2^ were used as the primary reinforcement. The nominal ply thickness was 0.15 mm, with warp and weft specifications of EC9 136 × 8.9 and EC9 136 × 6, respectively. The fiber density was 2.55 g/cm^3^.

The epoxy matrix system consisted of Tekno Marin ERA 4000-A epoxy resin and ERA 4000-B curing agent. These materials were selected due to their excellent compatibility with glass fiber fabrics, suitable wetting characteristics, and controlled curing behavior for hand lay-up processing. According to the manufacturer’s technical guidelines, the epoxy-to-curing agent ratio was maintained at 2:1 by weight to ensure consistent crosslinking density and reproducible mechanical performance.

Multi-walled carbon nanotubes (CNTs, 92% purity, diameter 8–10 nm), aluminum oxide (Al_2_O_3_, 99.99% purity, ~4 nm), and titanium dioxide (TiO_2_, 99.99% purity, ~17 nm) were incorporated as nanoscale modifiers. Nanoparticle contents were defined relative to the total resin mass to ensure consistent comparison across different formulations. CNT loadings 0.2, 0.5, and 2 wt% were selected to capture dispersion-controlled reinforcement and potential heterogeneity thresholds, while oxide nanoparticles were added at 2, 4, and 8 wt% to evaluate stiffness-driven reinforcement and embrittlement tendencies at elevated filler contents. The selected nanoparticle loading levels were determined based on both previous literature and the need to systematically capture the transition from dispersion-controlled reinforcement to heterogeneity-driven mechanical behavior. In CNT-reinforced polymer composites, these improvements are primarily attributed to effective dispersion, enhanced interfacial bonding, and nanoscale crack-bridging mechanisms [37,38,39]. However, further increasing CNT content typically results in agglomeration, localized stress concentration, and deterioration of mechanical properties [37,38].

Therefore, 0.2 wt% and 0.5 wt% CNTs were selected to represent the dispersion-controlled reinforcement regime, while 2 wt% was intentionally included to capture the onset of agglomeration-induced heterogeneity and mechanical degradation.

For oxide nanoparticles such as Al_2_O_3_ and TiO_2_, higher loading levels are generally required to produce measurable stiffness enhancement due to their particulate nature and lower aspect ratio. Previous studies have shown that moderate filler contents improve stiffness, whereas higher loadings promote elastic mismatch, particle clustering, and embrittlement effects. Accordingly, 2 wt%, 4 wt%, and 8 wt% were selected to systematically investigate the transition from moderate reinforcement to stiffness-dominated heterogeneity.

This multi-level loading strategy not only enables direct identification of the Composite Heterogeneity Threshold (CHT), but also provides a systematic framework for capturing the transition from homogeneous reinforcement to heterogeneity-dominated mechanical behavior.

Nanoparticles were dispersed into the epoxy resin via mechanical stirring (10 min) followed by probe-type ultrasonication (500 W, 20 kHz, 30 min) in pulse mode. The mixture temperature was maintained below 40 °C to prevent premature curing. Vacuum degassing was conducted for 15 min prior to hardener addition to reduce entrapped air.

Composite laminates were fabricated using a controlled hand lay-up process. Each ply was impregnated with nanoparticle-modified epoxy and consolidated using a steel roller to enhance fiber wetting and minimize void formation. The stacking sequence was arranged as [0/90]. Twelve plies were used for tensile and impact specimens, and eighteen plies for flexural specimens to satisfy span-to-thickness requirements. This fabrication approach follows the conventional hand lay-up methodology widely employed for woven fiber composite manufacturing [40].

Laminates were cured at room temperature for 24 h, followed by post-curing at 80 °C for 2 h to ensure complete crosslinking and stable mechanical performance. Fabrication parameters were kept constant for all composite systems to ensure that mechanical variations originated primarily from nanoparticle type and content rather than processing inconsistencies. Matrix formulations and nanoparticle loadings are summarized in Table 1.

For each composite formulation, five specimens were fabricated to ensure statistical reliability. Tensile tests were conducted using at least three valid measurements per condition, following standard practices where outlier values were excluded to ensure consistency. In contrast, flexural and impact tests were performed using five specimens for each formulation to capture variability more comprehensively. This approach ensures a balance between experimental accuracy and statistical representativeness.

### 2.2. Fiber Volume Fraction Estimation

Fiber volume fraction (V*f*) was estimated based on fabric areal density, ply count, measured laminate thickness, and constituent densities. Laminate thickness was measured using a calibrated digital micrometer at five different locations across each panel to minimize local thickness variation effects. The average measured thickness was 2.4–2.5 mm for 12-ply laminates and approximately 3.2 mm for 18-ply laminates.

The fiber volume fraction was calculated using the density-based relationship

$$Vf=\frac{nA_{f}}{\rho_{f}t}$$

where n is the number of plies, A_f_ is the areal density of the fabric, ρ_f_ is the fiber density (2.55 g/cm^3^), and *t* is the measured laminate thickness.

Based on these parameters, the estimated fiber volume fractions were:

- V*f* ≈ 0.38 for 12-ply laminates (tensile and impact specimens);
- V*f* ≈ 0.44 for 18-ply laminates (flexural specimens).

These values fall within the typical fiber volume fraction range (35–45%) reported for hand lay-up woven glass/epoxy laminates in the literature. Since nanoparticle incorporation modified only the matrix phase without altering fiber architecture or stacking sequence, variations in Vf among formulations are considered negligible.

Although direct matrix digestion (e.g., ASTM D3171) was not conducted, fabrication parameters—including probe-type ultrasonication, vacuum degassing, controlled resin-to-hardener ratio, and consistent manual consolidation—were kept constant for all laminate systems. Therefore, the presented V*f* values serve as consistent comparative indicators across formulations, and mechanical performance variations are attributed primarily to nanoparticle type and content rather than fiber volume inconsistencies.

### 2.3. Specimen Preparation

Test specimens were machined from cured laminates using CNC water-jet cutting to minimize thermal damage and edge-induced defects. Following cutting, specimen edges were lightly polished to remove potential surface irregularities and reduce premature crack initiation sites. Specimen dimensions were verified using calibrated digital instruments prior to testing.

A total of ten composite formulations (neat GF reference, CNT-modified, Al_2_O_3_-modified, and TiO_2_-modified systems at specified loadings) were investigated. For each formulation, five specimens were fabricated. Tensile test results were obtained from at least three valid measurements after excluding outliers, whereas flexural and impact tests were evaluated using five specimens. Reported mechanical values correspond to the mean of three independent measurements, and standard deviations were calculated to quantify experimental variability.

Specimen geometries were defined in accordance with the relevant ASTM standards:

- Tensile test: ASTM D3039 (250 mm × 25 mm × 2.5 mm). End tabs were bonded to specimen ends to prevent gripping damage and ensure uniform load transfer.
- Three-point bending test: ASTM D7264/D7264M (70 mm × 12.7 mm × 3.2 mm). Span length was selected to satisfy the recommended span-to-thickness ratio.
- Low-velocity impact test: ASTM D7136/D7136M (90 mm × 90 mm × 2.4 mm). Specimens were mounted using the standard fixture configuration to ensure consistent boundary conditions.

All specimens were conditioned under laboratory ambient conditions prior to testing to maintain consistent environmental exposure across formulations.

### 2.4. Mechanical Testing

Mechanical characterization was conducted under tensile, three-point bending, and low-velocity impact loading in accordance with the relevant ASTM standards. For each composite formulation, three independent specimens were tested (*n* = 3), and reported values correspond to mean results with calculated standard deviations.

#### 2.4.1. Tensile Test

Tensile tests were performed according to ASTM D3039 using an Instron 3367 universal testing machine equipped with a 100 kN load cell. Specimens (250 mm × 25 mm × 2.5 mm) were tested with a gauge length of 200 mm at a constant crosshead speed of 1 mm/min under ambient laboratory conditions.

Load and displacement data were continuously recorded. Engineering stress (σ) and strain (ε) were calculated as in the equation below.

It should be noted that strain values were calculated based on the crosshead displacement of the testing machine. Although this method may introduce certain inaccuracies due to system compliance, it was consistently applied to all specimens to ensure reliable comparative evaluation. Therefore, the results are primarily interpreted in a relative sense rather than as absolute strain values.

$$\sigma =\frac{F_{max}}{A}\;\;\varepsilon =\frac{L-L_{0}}{L_{0}}=\frac{\Delta L}{L_{0}}$$

where F is the applied load, A is the original cross-sectional area, L_0_ is the initial gauge length (200 mm), and ΔL is the crosshead displacement.

Tensile strength was defined as the maximum engineering stress prior to failure. The elastic modulus was determined from the initial linear region of the stress–strain curve using the automatic modulus calculation function in Instron Bluehill (version 3) software in accordance with ASTM D3039 guidelines.

#### 2.4.2. Three-Point Bending Test

Flexural properties were evaluated according to ASTM D7264/D7264M using a universal testing machine equipped with a 1 kN load cell. Specimens (70 mm × 12.7 mm × 3.2 mm) were tested with a support span of 51.2 mm, corresponding to a span-to-thickness ratio of 16:1 to promote bending-dominated deformation.

Flexural stress (σ_f_) and flexural strain (ε_f_) were calculated as:

$$\sigma_{f}=\frac{3FL}{2bh^{2}}\;\;{ԑ}_{f}=\frac{6h\delta }{L^{2}}$$

where F is the applied load, L is the support span, b is the specimen width, h is the specimen thickness, and δ is the mid-span deflection.

Flexural strength was defined as the maximum stress prior to failure. The flexural modulus was determined from the initial linear portion of the stress–deflection response.

#### 2.4.3. Low-Velocity Impact Test

Low-velocity impact tests were conducted in accordance with ASTM D7136/D7136M using an instrumented drop-weight impact system. A hemispherical impactor with a mass of 5.5 kg and a radius of 10 mm was employed. The drop height was adjusted to achieve an impact velocity of approximately 2.7 m/s, corresponding to an initial impact energy of ~20 J. The selected impact energy level (~20 J) was intended to induce controlled damage without full penetration. Post-impact observations indicated that the specimens predominantly exhibited barely visible impact damage (BVID), rather than complete perforation. This damage regime enables evaluation of progressive damage evolution and energy absorption mechanisms under sub-critical impact conditions. Specimens were mounted using the standard clamped fixture configuration specified by ASTM D7136 to ensure consistent boundary conditions. Force–time and force–displacement data were continuously recorded during impact.

The absorbed impact energy (E_abs_) was calculated from the area under the force–displacement curve:

Impact strength (IS) was determined by normalizing the absorbed energy with respect to specimen thickness.

### 2.5. Fracture Surface Analysis

Fracture surfaces of tensile and impact specimens were examined using scanning electron microscopy (SEM) to investigate microstructural damage mechanisms and interfacial characteristics. Prior to observation, fractured specimens were sputter-coated with a thin conductive gold layer to prevent surface charging. SEM analyses were conducted under high-vacuum conditions at accelerating voltages between 10–20 kV.

Microstructural evaluation focused on identifying dominant failure mechanisms, including matrix cracking, fiber pull-out, interfacial debonding, crack deflection, and nanoparticle agglomeration. Observations were performed at multiple magnifications (100×–2000×) to assess fiber–matrix interfacial integrity, crack propagation paths, and dispersion quality of nanoscale reinforcements.

Elemental analysis and mapping were conducted using an energy-dispersive X-ray spectroscopy (EDS) system integrated with the SEM. Spectra and elemental maps were acquired under identical operating parameters to ensure comparability among composite formulations. For CNT-modified systems, dispersion quality was primarily evaluated through spatial uniformity rather than absolute carbon intensity due to the carbon-rich polymer matrix. For oxide-reinforced systems, aluminum (Al) and titanium (Ti) elemental signals were used to assess nanoparticle distribution and interfacial localization.

Particular emphasis was placed on correlating fracture morphology and elemental distribution patterns with mechanical performance trends, enabling microstructure–property linkage and supporting identification of the proposed Composite Heterogeneity Threshold (CHT).

### 2.6. Finite Element Modeling

Finite element modeling of composite materials has been widely applied to predict damage evolution and structural response under complex loading conditions [41,42]. Finite element simulations were performed in ANSYS LS-DYNA using the MAT_022 composite damage model under quasi-static solution conditions. A displacement-controlled loading configuration was adopted to replicate the experimental tensile, flexural, and impact boundary conditions while ensuring numerical stability.

Composite laminates were modeled as homogenized orthotropic continua with effective elastic properties derived directly from experimental tensile responses (Table 2). Owing to the balanced 0°/90° woven glass architecture, in-plane stiffness symmetry was assumed (E_1_ = E_2_).

Although direct experimental characterization of through-thickness elastic properties was not performed, the corresponding out-of-plane modulus (E_z_) and shear components (G_xz_, G_yz_) were approximated using in-plane properties to maintain internal consistency within the homogenized orthotropic framework. This assumption does not imply full three-dimensional isotropy; rather, it represents an effective stiffness idealization aimed at capturing global structural responses under tensile, flexural, and impact loading conditions.

Since the objective of the numerical model is comparative multi-formulation analysis and global stiffness–strength trend evaluation rather than detailed interlaminar stress prediction or delamination modeling, the adopted elastic symmetry provides a mechanically consistent and computationally stable representation of the woven laminate systems.

Strength parameters required by MAT_022—including longitudinal and transverse tensile strengths (XT, YT) and in-plane shear strength (SC)—were defined based on experimentally obtained mechanical data. Compressive strengths (XC, YC) were estimated using tensile and flexural responses to ensure internal mechanical consistency within the orthotropic continuum formulation.

Specimen geometries replicated the experimental configurations and were discretized using three-dimensional solid elements. The numerical models were discretized using structured hexahedral elements to ensure stable stress distribution and computational efficiency. The selected mesh density was determined based on preliminary sensitivity considerations, where further refinement resulted in negligible changes in global response parameters such as peak stress and stiffness. Therefore, the adopted mesh configuration is considered sufficient to ensure mesh-independent and reliable simulation results within the scope of the present study.

Boundary conditions were defined to match the respective ASTM test setups, including support constraints and contact definitions for bending and impact simulations. In the three-point bending simulations, contact interactions between the indenter, support rollers, and composite specimen were explicitly defined using a surface-to-surface contact algorithm. The contact behavior was modeled considering normal contact stiffness to prevent excessive penetration, while a constant friction coefficient was included to represent realistic interface interaction conditions.

Similarly, in impact simulations, the interaction between the hemispherical impactor and the composite laminate was defined using automatic contact formulations within LS-DYNA, ensuring stable force transfer and accurate representation of transient contact behavior. Boundary constraints were applied to replicate the clamped fixture configuration used in experimental impact testing.

These definitions ensure that the numerical model closely represents the experimental loading conditions and enhances the reliability of the simulation results.

**Figure 1:**
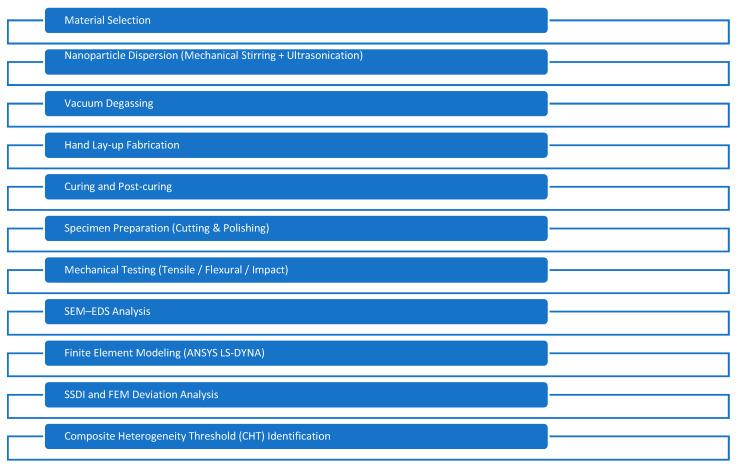
Methodology flowchart illustrating the experimental and numerical procedure used in this study.

The adopted homogenized modeling approach captures global stiffness evolution and strength-driven degradation trends. However, nanoparticle-scale heterogeneity, interfacial decohesion, and explicit crack-path propagation were not modeled directly. Therefore, deviations between experimental and numerical peak responses are interpreted within the proposed Composite Heterogeneity Threshold (CHT) framework as physically meaningful indicators of dispersion-induced microstructural heterogeneity rather than modeling inaccuracies. The overall experimental–numerical methodology is illustrated in Figure 1.

## 3. Results and Discussion

The mechanical response of nanoparticle-modified woven glass/epoxy composites is governed by the interplay between interfacial load transfer efficiency, dispersion stability, and elastic heterogeneity. In order to establish a unified reinforcement mechanism across tensile, flexural, and impact loading, the experimental results are first evaluated independently for each loading mode, followed by a microstructural correlation based on SEM–EDS observations. Finally, numerical predictions are interpreted within the framework of a Critical Heterogeneity Threshold (CHT), defined as the nanoparticle concentration beyond which stiffness enhancement no longer translates into proportional strength or toughness improvement due to localized elastic mismatch and dispersion-induced stress amplification.

### 3.1. Elastic Stiffness Evolution and Load Transfer Efficiency

**Figure 2:**
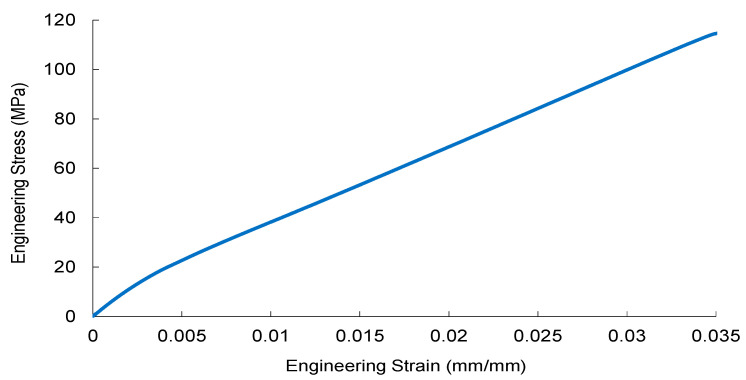
The engineering stress–strain curve of the reference glass fiber (GF) composite.

**Figure 3:**
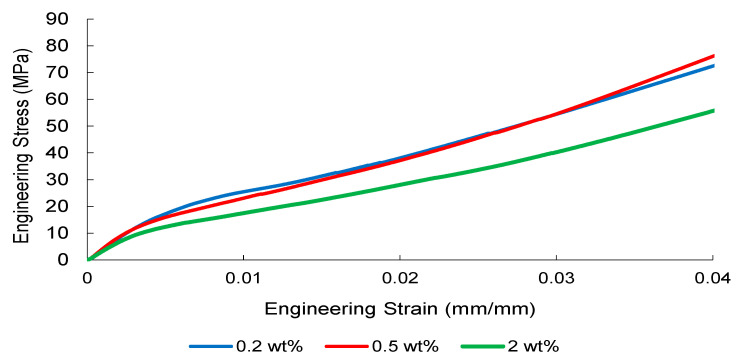
Engineering stress–strain curves of CNT-reinforced composite obtained from three independent tensile tests.

**Figure 4:**
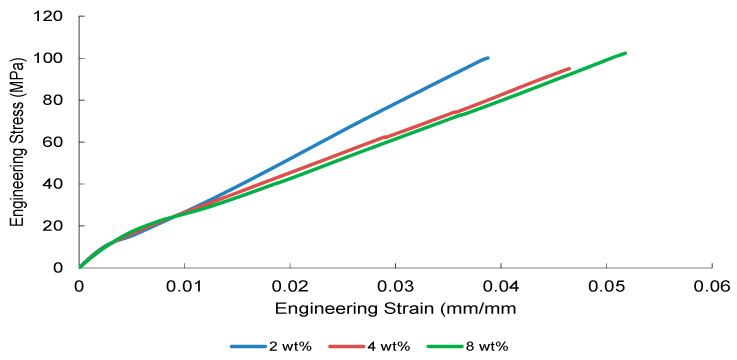
Engineering stress–strain curves of Al_2_O_3_-reinforced composite obtained from three independent tensile tests.

**Figure 5:**
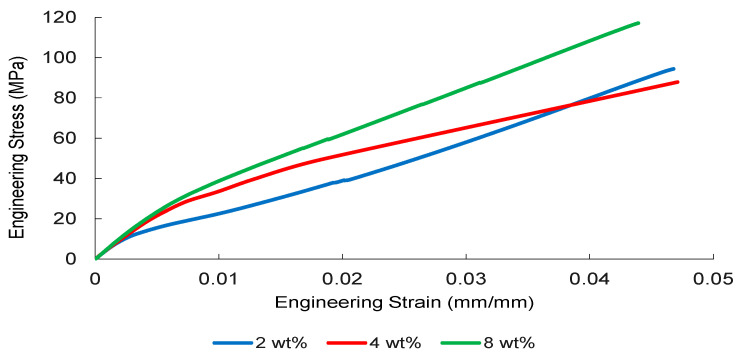
Engineering stress–strain curves of the TiO_2_-reinforced composite obtained from three independent tensile tests.

The tensile stress–strain responses of nanoparticle-modified woven glass/epoxy composites reveal a reinforcement mechanism governed primarily by elastic load transfer efficiency and dispersion stability. Representative engineering stress–strain curves are presented in Figure 2, Figure 3, Figure 4 and Figure 5.

As shown in Figure 2, the reference GF laminate exhibits a stable and nearly linear elastic regime following the initial matrix-dominated nonlinear region. The consistent slope indicates efficient stress transfer across the fiber–matrix interface and confirms that load bearing is dominated by the continuous glass fiber architecture.

CNT-modified systems (Figure 3) display a clear concentration-dependent evolution within the elastic region. The 0.5 wt% CNT formulation demonstrates the steepest initial slope, suggesting enhanced interfacial shear transfer and effective crack-bridging within the fiber–matrix network. In contrast, the 0.2 wt% system provides only marginal stiffness enhancement, implying insufficient nanotube network formation. Increasing CNT loading to 2 wt% does not further improve the elastic slope; instead, curve smoothness deteriorates, consistent with agglomeration-induced local stiffness heterogeneity that limits efficient load redistribution. This behavior can be attributed to nanoscale stress transfer efficiency and interfacial shear strengthening. At optimal dispersion, nanoparticles enhance load bridging between fiber and matrix, delaying microcrack initiation. However, as particle agglomeration increases, local stiffness gradients emerge, leading to stress concentration zones that disrupt uniform load transfer and initiate premature damage.

Al_2_O_3_-reinforced composites (Figure 4) exhibit a stiffness-dominated response, with the 2 wt% formulation achieving the most stable elastic slope. Higher loadings (4 and 8 wt%) show reduced slope consistency, suggesting the onset of particle clustering and elastic mismatch amplification at the particle–matrix interface.

TiO_2_-modified systems (Figure 5) present a more progressive stiffness evolution. The 8 wt% formulation maintains a relatively steep and stable elastic regime without abrupt instability, indicating that within the investigated concentration range, dispersion-induced heterogeneity has not yet resulted in severe macroscopic stress localization.

Collectively, Figure 2, Figure 3, Figure 4 and Figure 5 demonstrate that elastic stiffness evolution is not solely governed by filler fraction but by the interplay between dispersion uniformity and interfacial load transfer efficiency. These observations suggest the presence of a reinforcement window preceding a dispersion-induced heterogeneity transition, which is further quantified in the subsequent sections.

### 3.2. Strength Evolution and Reinforcement Window

**Figure 6:**
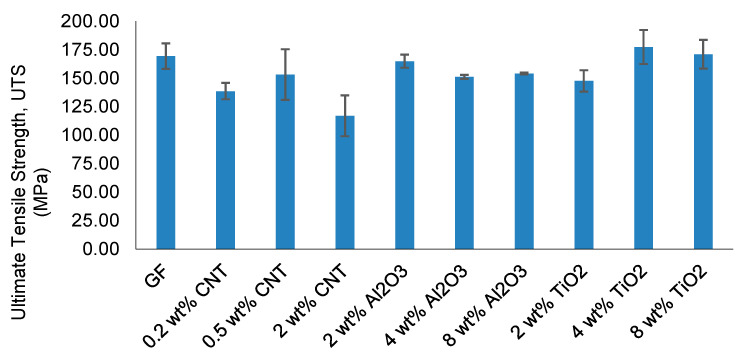
Ultimate tensile strength of CNT-, Al_2_O_3_-, and TiO_2_-modified woven glass/epoxy composites (mean ± SD).

**Figure 7:**
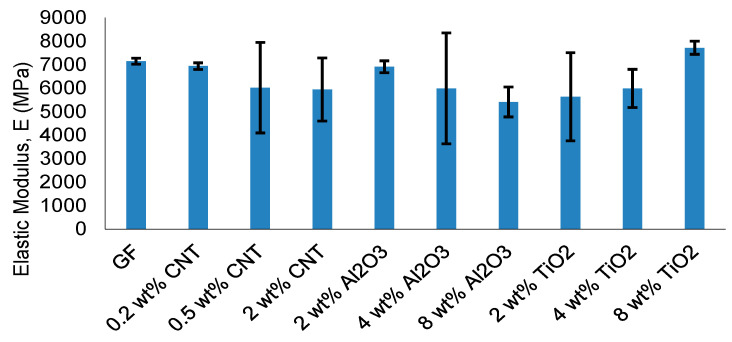
Mean elastic modulus (±SD) of GF, CNT-, Al_2_O_3_-, and TiO_2_-reinforced composites (*n* = 3).

The evolution of ultimate tensile strength (UTS) and elastic modulus provides quantitative insight into the reinforcement efficiency and the emergence of stiffness–strength decoupling. The corresponding results are summarized in Figure 6 and Figure 7.

As shown in Figure 6, CNT-modified systems exhibit a non-monotonic strength evolution. The 0.5 wt% CNT formulation achieves the highest UTS among all CNT concentrations, indicating an optimal dispersion state that maximizes interfacial shear transfer and crack-bridging efficiency. Increasing CNT content to 2 wt% results in a measurable reduction in tensile strength accompanied by higher standard deviation, consistent with agglomeration-induced microstructural heterogeneity and localized stress amplification. Mechanistically, this transition reflects the competition between crack-bridging and stress concentration effects. While well-dispersed nanoparticles promote energy dissipation through crack deflection and interfacial strengthening, agglomerated particles act as micro-defects, accelerating crack initiation and reducing effective load-bearing capacity.

Al_2_O_3_-reinforced composites display a stiffness-dominated reinforcement trend. Strength increases at 2 wt%, confirming effective particle dispersion and load sharing. However, further increasing filler content to 4 and 8 wt% leads to progressive strength reduction, suggesting excessive local rigidity and interfacial strain incompatibility that promote premature microcrack initiation.

TiO_2_-modified systems demonstrate moderate strength enhancement at lower concentrations, followed by systematic degradation at higher loadings. The decline in UTS at elevated TiO_2_ content reflects increased elastic mismatch and reduced interfacial stability despite continued stiffness contribution from ceramic inclusions.

To provide a mechanistic interpretation of these trends, the observed non-monotonic tensile strength evolution is primarily governed by nanoparticle dispersion quality rather than filler content alone. Similar non-monotonic trends have been widely reported in nanoparticle-reinforced polymer composites. Previous studies have shown that while low nanoparticle contents improve load transfer efficiency and crack-bridging behavior, higher concentrations often lead to agglomeration, resulting in localized stress concentrations and premature failure [43,44]. In particular, CNT-reinforced epoxy systems exhibit optimal mechanical performance at low weight fractions, beyond which dispersion quality deteriorates significantly [45]. Likewise, oxide nanoparticles such as Al_2_O_3_ and TiO_2_ have been reported to enhance stiffness while showing limited or inconsistent strength improvement due to interfacial incompatibility and particle clustering at higher loadings [29,46]. These findings are in strong agreement with the present results, confirming that nanoparticle reinforcement is fundamentally governed by dispersion stability and interfacial integrity rather than filler concentration alone.

Similarly, oxide nanoparticle systems (Al_2_O_3_ and TiO_2_) exhibit a dispersion-sensitive response rather than a purely stiffness-driven enhancement. While moderate improvements are observed at lower filler contents, the absence of consistent strength enhancement at higher loadings indicates that interfacial compatibility and particle distribution dominate mechanical performance. These observations confirm that nanoparticle reinforcement in woven glass/epoxy composites is not solely concentration-dependent but is critically governed by dispersion-induced heterogeneity, which defines the effective reinforcement limit and marks the onset of the Composite Heterogeneity Threshold (CHT).

Beyond this threshold, further nanoparticle addition does not contribute to effective reinforcement but instead amplifies structural inefficiencies arising from dispersion instability, marking the practical boundary of reinforcement effectiveness. This distinction is critical, as it demonstrates that the observed deviations are not deficiencies of the numerical model, but rather signatures of dispersion-controlled microstructural transitions that cannot be captured within classical homogenization frameworks.

The elastic modulus trends shown in Figure 7 reveal a different evolution pattern. While moderate nanoparticle loading enhances stiffness across most systems, modulus improvement does not consistently translate into proportional strength gains. In particular, modulus can remain elevated even when UTS decreases, indicating the onset of stiffness–strength decoupling.

Collectively, the divergence between strength and stiffness trends defines a reinforcement window within which mechanical enhancement remains dispersion-controlled. Beyond this window, stiffness enhancement persists but tensile strength deteriorates due to heterogeneity-driven stress localization. This transition marks the mechanical boundary later formalized as the Composite Heterogeneity Threshold (CHT).

### 3.3. Quantitative Identification of the Composite Heterogeneity Threshold (CHT)

The divergence between tensile strength and elastic modulus trends indicates that reinforcement efficiency cannot be evaluated solely through stiffness enhancement. To quantitatively capture the onset of stiffness–strength decoupling, a Strength–Stiffness Decoupling Index (SSDI) was introduced. From a physical perspective, the CHT represents a transition from a homogeneous stress transfer regime to a heterogeneity-dominated mechanical response. Below this threshold, the composite behaves as an effectively continuous medium with stable load redistribution. Beyond this point, local stiffness mismatches and interfacial discontinuities govern damage evolution, leading to reduced mechanical efficiency and increased model–experiment deviation.

The SSDI is defined as:

$$\mathrm{SSDI}\;=\;\frac{\left(\frac{E}{E_{0}}\right)}{\left(\frac{\sigma }{\sigma_{0}}\right)}$$

**Figure 8:**
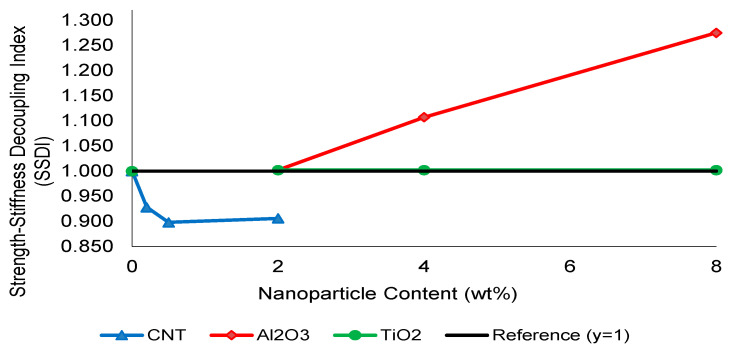
Strength–Stiffness Decoupling Index (SSDI) as a function of nanoparticle type and content.

SSDI is a dimensionless parameter designed to capture the imbalance between stiffness and strength evolution, where E and σ denote the elastic modulus and ultimate tensile strength of the nanoparticle-modified composite, respectively, while E_0_ and σ_0_ correspond to those of the reference GF laminate. An SSDI value equal to unity indicates proportional stiffness and strength enhancement, whereas deviations from unity reflect the onset of stiffness–strength decoupling and microstructural heterogeneity. Values below unity reflect strength-dominated reinforcement, whereas SSDI > 1 indicates stiffness-driven response without proportional strength benefit, signifying the onset of stiffness–strength decoupling. As illustrated in Figure 8, CNT-modified systems remain within the efficient reinforcement regime (SSDI ≈ 1) at 0.5 wt%, confirming proportional stiffness and strength enhancement. However, at higher loadings, SSDI deviates from unity, indicating increasing dispersion-induced heterogeneity.

Al_2_O_3_-reinforced systems exhibit a pronounced increase in SSDI beyond moderate filler levels, clearly exceeding unity and marking the transition toward stiffness-dominated embrittlement. TiO_2_ systems demonstrate a more gradual evolution, remaining closer to unity within the investigated concentration range.

Based on this quantitative framework, the Composite Heterogeneity Threshold (CHT) is defined as the nanoparticle concentration at which SSDI first exceeds unity, indicating that further stiffness enhancement no longer translates into proportional strength improvement. This threshold represents the transition from dispersion-controlled reinforcement to heterogeneity-dominated mechanical behavior.

To further validate this boundary, an FEM deviation index (η) was introduced:

$$\eta \;=\;\frac{(\sigma_{exp}-\sigma_{FEM})\;}{\sigma_{exp}\times 100}$$

where σ_exp_ and σ_FEM_ denote the experimental and numerical tensile strengths, respectively. This index quantifies the relative deviation between experimental measurements and FEM predictions.

For example, for the 0.5 wt% CNT composite, the deviation between experimental and FEM tensile strengths remains very low (η ≈ 0.41%), indicating strong agreement between experimental and numerical results within the optimal dispersion regime.

While SSDI and the FEM deviation index (η) quantitatively capture stiffness–strength decoupling and model–experiment divergence, these scalar metrics alone cannot fully resolve the underlying microstructural mechanisms. Therefore, SEM and EDS analyses were employed to directly investigate fracture morphology, nanoparticle dispersion stability, and interfacial integrity. The microstructural evidence demonstrates that beyond the optimal reinforcement regime, dispersion-induced heterogeneity, localized stress concentrations, and interfacial debonding become dominant. This multi-scale correlation provides direct physical validation of the Composite Heterogeneity Threshold (CHT), establishing it not only as a numerical boundary but as a microstructure-governed transition in load transfer efficiency and damage evolution.

Overall, the proposed Composite Heterogeneity Threshold (CHT) framework provides a unified mechanistic and quantitative interpretation of nanoparticle reinforcement in woven glass fiber composites. By integrating experimental observations, SSDI-based stiffness–strength decoupling analysis, and FEM deviation metrics, the present study establishes a direct link between nanoparticle dispersion stability, load transfer efficiency, and model reliability. Unlike conventional approaches that evaluate reinforcement solely based on strength or stiffness improvements, the CHT concept defines a physically meaningful transition boundary beyond which additional nanoparticle content becomes detrimental rather than beneficial. This framework not only advances the fundamental understanding of dispersion-controlled mechanical behavior but also offers practical design guidelines for optimizing nanoparticle loading in high-performance composite systems.

### 3.4. Microstructural Validation of the Composite Heterogeneity Threshold (CHT)

Scanning electron microscopy (SEM) and energy-dispersive spectroscopy (EDS) analyses (Figure 9, Figure 10, Figure 11, Figure 12, Figure 13, Figure 14, Figure 15, Figure 16, Figure 17, Figure 18, Figure 19, Figure 20, Figure 21, Figure 22, Figure 23, Figure 24 and Figure 25) provide direct microstructural evidence supporting the mechanically defined Composite Heterogeneity Threshold (CHT). Fracture morphologies reveal a dispersion-controlled reinforcement window preceding the onset of heterogeneity-dominated failure.

#### 3.4.1. Reference GF: Baseline Interfacial Failure

**Figure 9:**
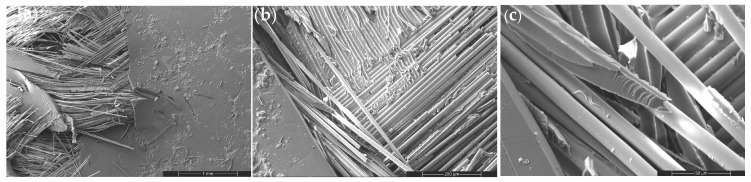
SEM micrographs of the tensile fracture surface of the neat woven GF composite at increasing magnifications: (**a**) 100×, (**b**) 500×, and (**c**) 2000×.

The neat GF composite (Figure 9) exhibits an interfacial debonding-dominated failure mechanism characterized by matrix cracking, extensive fiber pull-out, and smooth fiber surfaces. The absence of catastrophic fiber rupture indicates that failure is governed by interface-controlled load transfer rather than intrinsic fiber strength. High-magnification images confirm frictional sliding at the fiber–matrix interface, establishing the interphase region as the critical determinant of tensile failure. This baseline fracture morphology provides a reference for evaluating nanoparticle-induced modifications in stress redistribution behavior.

#### 3.4.2. CNT Systems: Dispersion-Controlled Toughening

**Figure 10:**
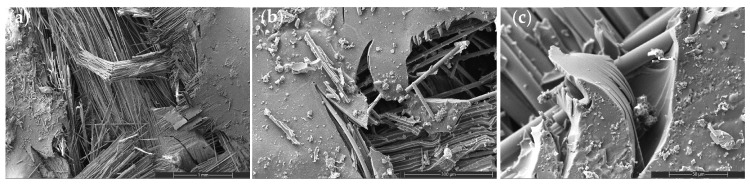
SEM images of the tensile fracture surface of the 0.2 wt% CNT-reinforced composite at different magnifications: (**a**) 100×, (**b**) 500×, and (**c**) 2000×.

**Figure 11:**
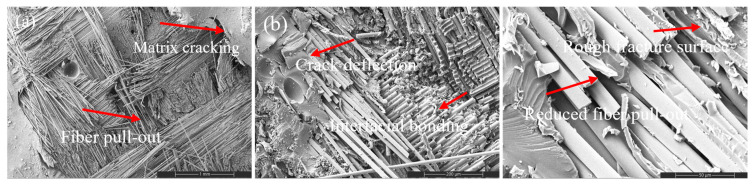
SEM images of the tensile fracture surface of the 0.5 wt% CNT-reinforced composite at different magnifications: (**a**) 100×, (**b**) 500×, and (**c**) 2000×.

**Figure 12:**
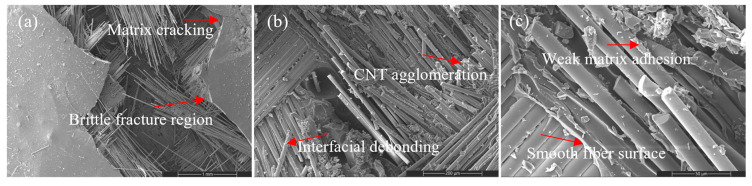
SEM images of the tensile fracture surface of the 2 wt% CNT-reinforced composite at different magnifications: (**a**) 100×, (**b**) 500×, and (**c**) 2000×.

CNT incorporation introduces a dispersion-sensitive toughening mechanism (Figure 10, Figure 11 and Figure 12). At low CNT loading (0.2 wt%), fracture surfaces exhibit mixed-mode behavior characterized by localized matrix cracking and limited nanotube bridging. The presence of short bridging zones indicates partial stress redistribution without significant toughening.

At moderate loading (0.5 wt%), improved dispersion promotes pronounced crack deflection, reduced clean fiber pull-out length, and increased surface roughness (Figure 11). The fracture path becomes more tortuous, reflecting enhanced interfacial shear resistance and distributed stress transfer. This morphology directly correlates with the peak tensile, flexural, and impact performance observed in Section 3.2.

However, at higher CNT content (2 wt%), nanoparticle agglomeration introduces localized stress concentration sites and smoother fiber surfaces (Figure 12). Crack deflection diminishes and interfacial debonding becomes dominant, signaling reduced toughening efficiency. This morphological transition coincides with SSDI deviation and confirms the onset of the CHT.

Thus, CNT reinforcement operates within a narrow dispersion-controlled window in which crack-bridging and stress redistribution mechanisms are maximized without triggering microstructural instability.

#### 3.4.3. Al_2_O_3_ Systems: Stiffness–Toughness Transition

**Figure 13:**
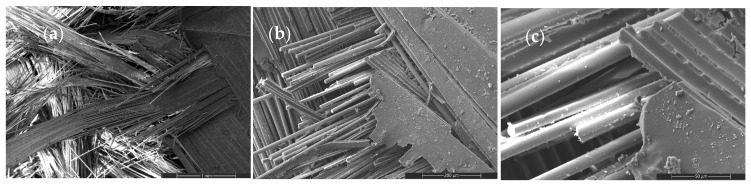
SEM images of the tensile fracture surface of the 2 wt% Al_2_O_3_-reinforced composite at different magnifications: (**a**) 100×, (**b**) 500×, and (**c**) 2000×.

**Figure 14:**
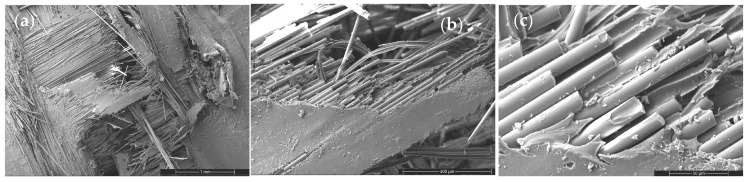
SEM images of the tensile fracture surface of the 4 wt% Al_2_O_3_-reinforced composite at different magnifications: (**a**) 100×, (**b**) 500×, and (**c**) 2000×.

Al_2_O_3_ nanoparticles introduce a stiffness-dominated reinforcement mechanism (Figure 12, Figure 13 and Figure 14). At moderate loading (2 wt%), fracture surfaces appear compact with controlled crack deflection and limited large-scale pull-out, indicating improved matrix constraint.

At intermediate loading (4 wt%), a balanced failure mode combining controlled fiber fracture and moderate pull-out is observed, corresponding to optimal stiffness–strength synergy.

**Figure 15:**
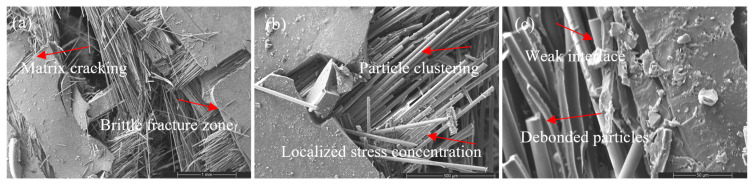
SEM images of the tensile fracture surface of the 8 wt% Al_2_O_3_-reinforced composite at different magnifications: (**a**) 100×, (**b**) 500×, and (**c**) 2000×.

At high loading (8 wt%), particle clustering and interfacial discontinuities promote microvoid formation and brittle fracture features (Figure 15). The failure mode shifts toward interface-dominated fracture with reduced energy dissipation capacity. This morphology confirms that excessive stiffness amplification leads to embrittlement once dispersion uniformity deteriorates, consistent with the stiffness-driven regime of the CHT.

#### 3.4.4. TiO_2_ Systems: Progressive Stress Redistribution

**Figure 16:**
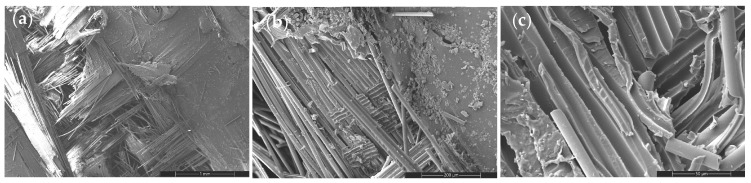
SEM images of the tensile fracture surface of the 2 wt% TiO_2_-reinforced composite at different magnifications: (**a**) 100×, (**b**) 500×, and (**c**) 2000×.

**Figure 17:**
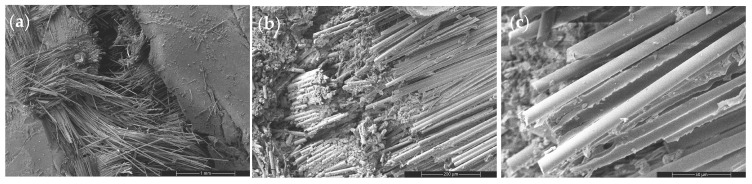
SEM images of the tensile fracture surface of the 4 wt% TiO_2_-reinforced composite at different magnifications: (**a**) 100×, (**b**) 500×, and (**c**) 2000×.

**Figure 18:**
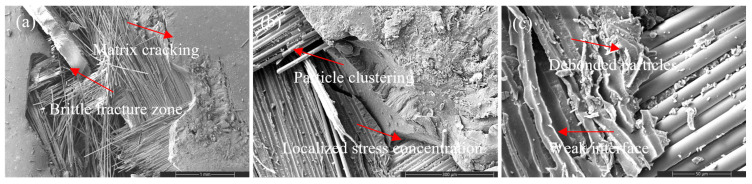
SEM images of the tensile fracture surface of the 8 wt% TiO_2_-reinforced composite at different magnifications: (**a**) 100×, (**b**) 500×, and (**c**) 2000×.

TiO_2_ modification alters fracture behavior through particle-induced stress redistribution and interfacial interaction mechanisms (Figure 16, Figure 17 and Figure 18).

TiO_2_ modification produces a more gradual fracture evolution. At 2–4 wt%, mixed-mode cracking and controlled matrix shear deformation dominate, indicating effective stress redistribution and stable interfacial bonding.

At 8 wt%, fracture morphology becomes more compact and brittle, with shorter pull-out lengths and localized stress amplification regions. Compared to CNT systems, TiO_2_ exhibits weaker crack-bridging efficiency but a smoother transition toward heterogeneity-dominated behavior. This progressive evolution reinforces the microstructural basis of the CHT framework.

This evolution reflects a progressive transition from distributed stress redistribution to elastic mismatch-dominated failure, reinforcing the microstructural basis of the CHT.

### 3.5. Elemental Mapping and Dispersion Stability Assessment

#### 3.5.1. Reference GF: Baseline Chemical Stability

**Figure 19:**
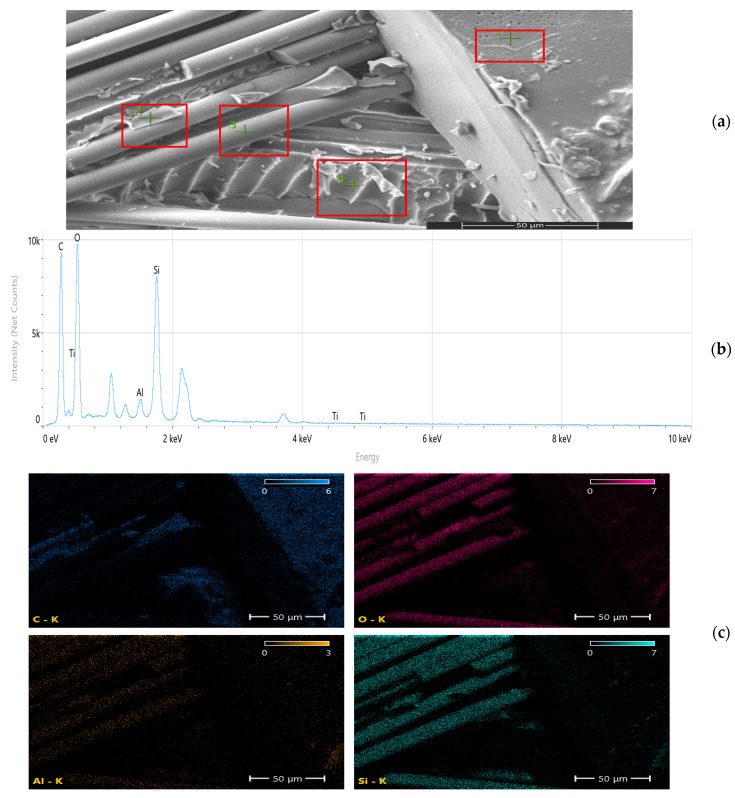
Microstructural and elemental characterization of the GF composite: (**a**) SEM fracture morphology, (**b**) EDS spectrum, (**c**) elemental mapping.

The reference GF laminate exhibits a chemically stable two-phase architecture, confirmed by EDS spectra and elemental mapping (Figure 19, Table 3). Carbon-rich domains correspond to the polymer matrix, while silicon-enriched regions mark the glass fibers. The spatial segregation remains sharp yet continuous, indicating stable interfacial integrity and efficient load transfer dominated by shear resistance rather than fiber rupture. This baseline condition establishes the homogeneous reference state against which nanoparticle-induced heterogeneity is evaluated.

#### 3.5.2. CNT Systems: Carbon Network Evolution and Heterogeneity Onset

**Figure 20:**
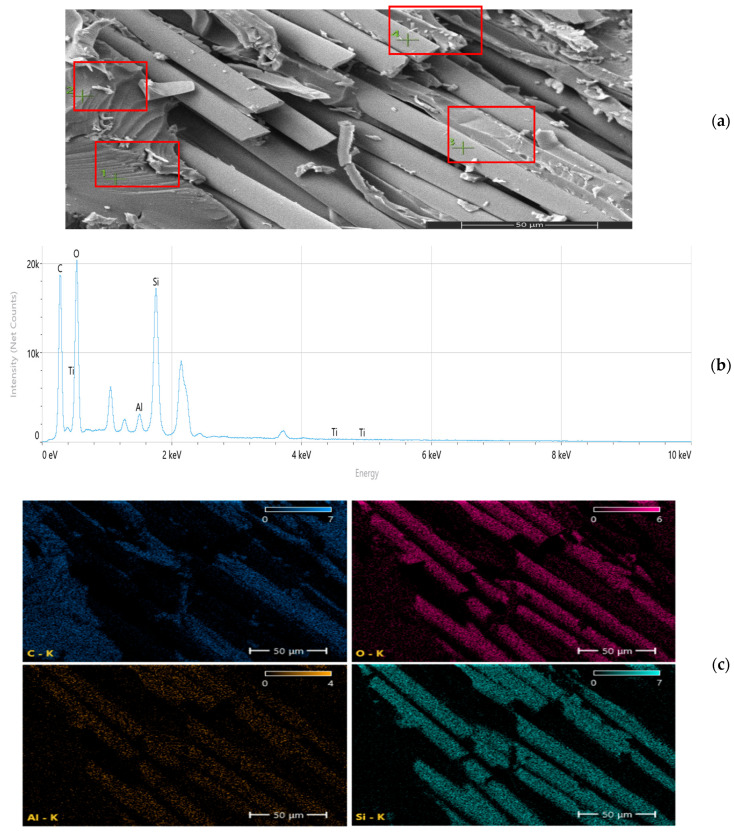
Microstructural and elemental characterization of the 0.5 wt% CNT composite: (**a**) SEM fracture morphology, (**b**) EDS spectrum, (**c**) elemental mapping.

At 0.5 wt% CNTs (Figure 20, Table 4), elemental mapping reveals a relatively uniform carbon distribution with minimal spatial intensity gradients. This condition coincides with maximum mechanical performance and negligible FEM deviation, indicating effective stress redistribution through a percolated nanoscale network.

**Figure 21:**
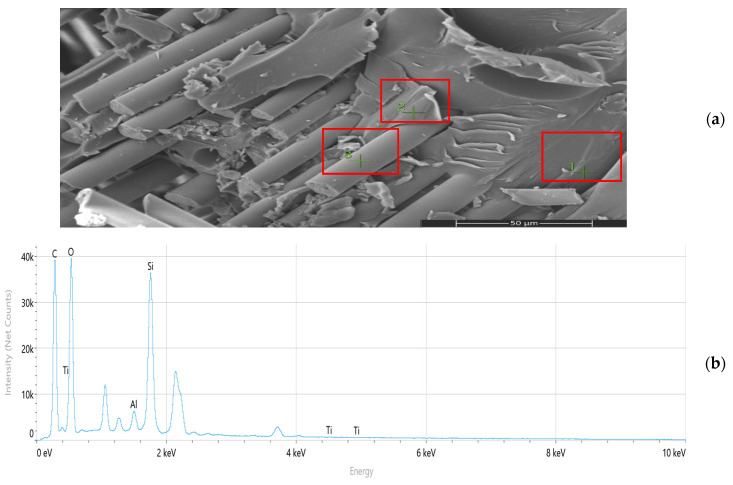

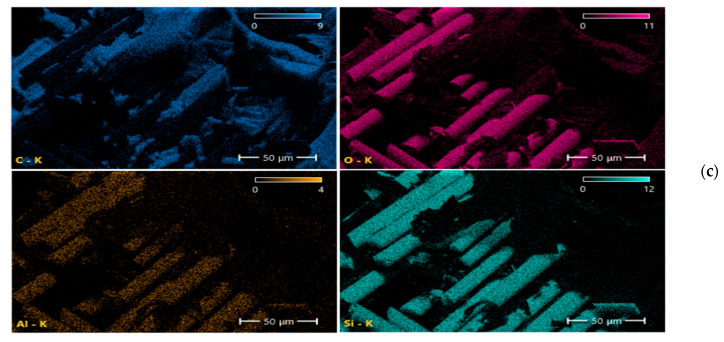
Microstructural and elemental characterization of the 2 wt% CNT composite: (**a**) SEM fracture morphology, (**b**) EDS spectrum, (**c**) elemental mapping.

However, at 2 wt% CNTs (Figure 21, Table 5), pronounced spatial carbon intensity fluctuations emerge. Localized carbon-enriched clusters introduce stiffness gradients and interfacial stress concentration zones. This marks the microstructural onset of the Composite Heterogeneity Threshold (CHT), beyond which homogenized continuum assumptions progressively lose validity.

#### 3.5.3. Al_2_O_3_ Systems: Ceramic Clustering and Elastic Contrast Amplification

**Figure 22:**
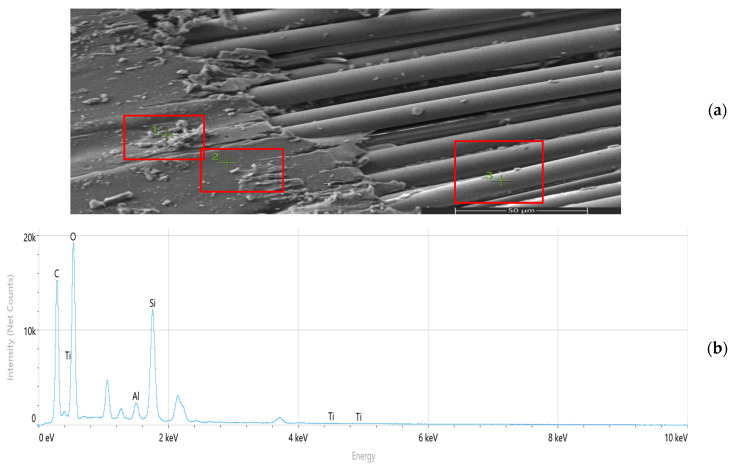

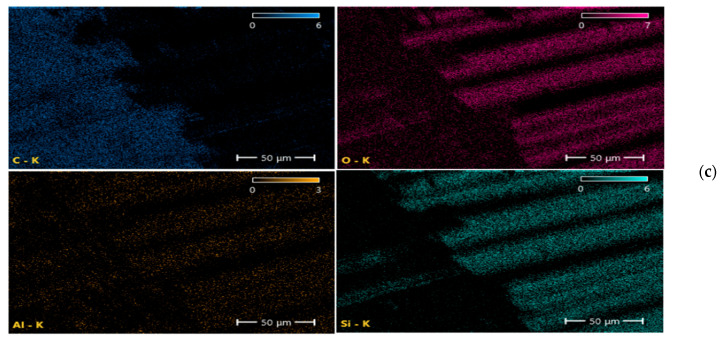
Microstructural and elemental characterization of the 2 wt% Al_2_O_3_ composite: (**a**) SEM fracture morphology, (**b**) EDS spectrum, (**c**) elemental mapping.

At 2 wt% Al_2_O_3_ (Figure 22, Table 6), aluminum mapping remains relatively dispersed, suggesting moderate elastic mismatch without severe clustering. The fracture surface indicates controlled particle–matrix interaction.

**Figure 23:**
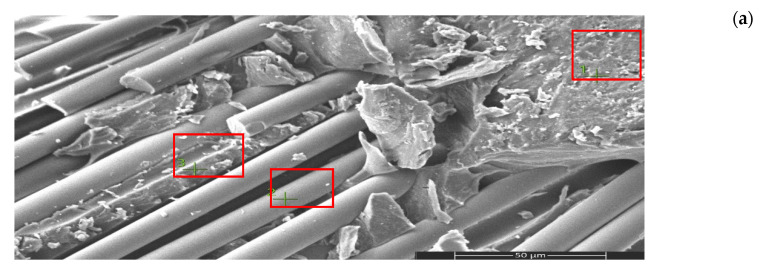

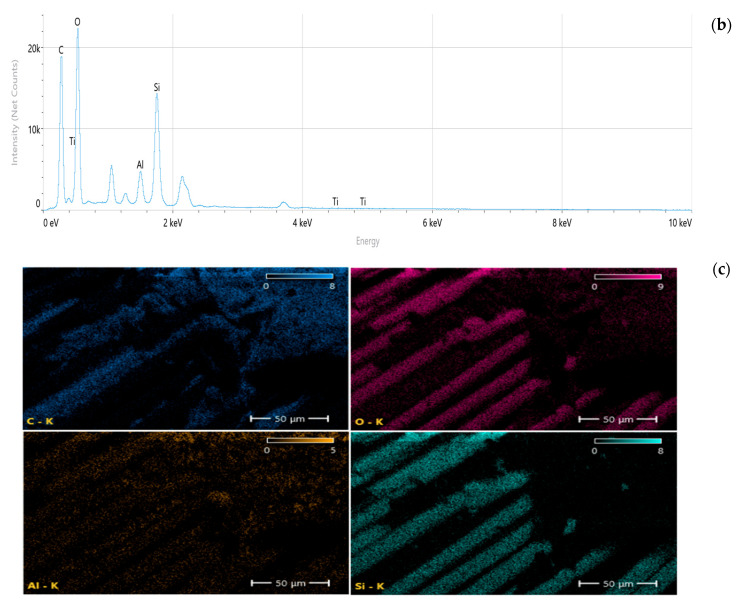
Microstructural and elemental characterization of the 8 wt% Al_2_O_3_ composite: (**a**) SEM fracture morphology, (**b**) EDS spectrum, (**c**) elemental mapping.

At 8 wt% Al_2_O_3_ (Figure 23, Table 7), particle-rich agglomerated regions become evident. Elemental gradients intensify, correlating with amplified stiffness mismatch and reduced fracture resistance. The spatial aluminum concentration gradients directly correspond to the increased FEM deviation observed at higher filler contents.

#### 3.5.4. TiO_2_ Systems: Progressive Heterogeneity Development

**Figure 24:**
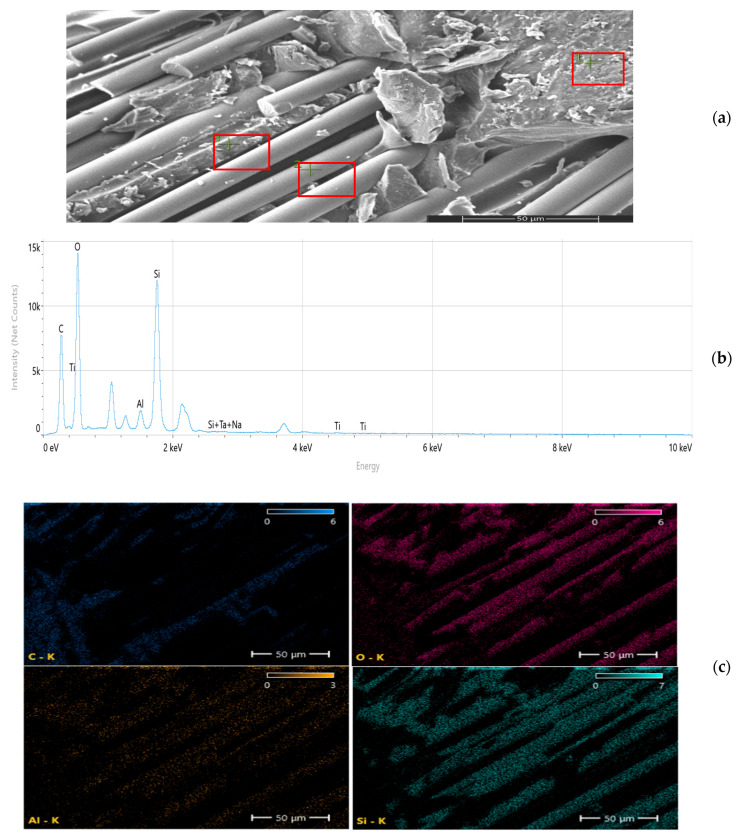
Microstructural and elemental characterization of the 2 wt% TiO_2_ composite: (**a**) SEM fracture morphology, (**b**) EDS spectrum, (**c**) elemental mapping.

TiO_2_ systems demonstrate a gradual rather than abrupt dispersion transition. At 2 wt% (Figure 24, Table 8), Ti distribution remains relatively uniform, with limited clustering and moderate interfacial compatibility.

**Figure 25:**
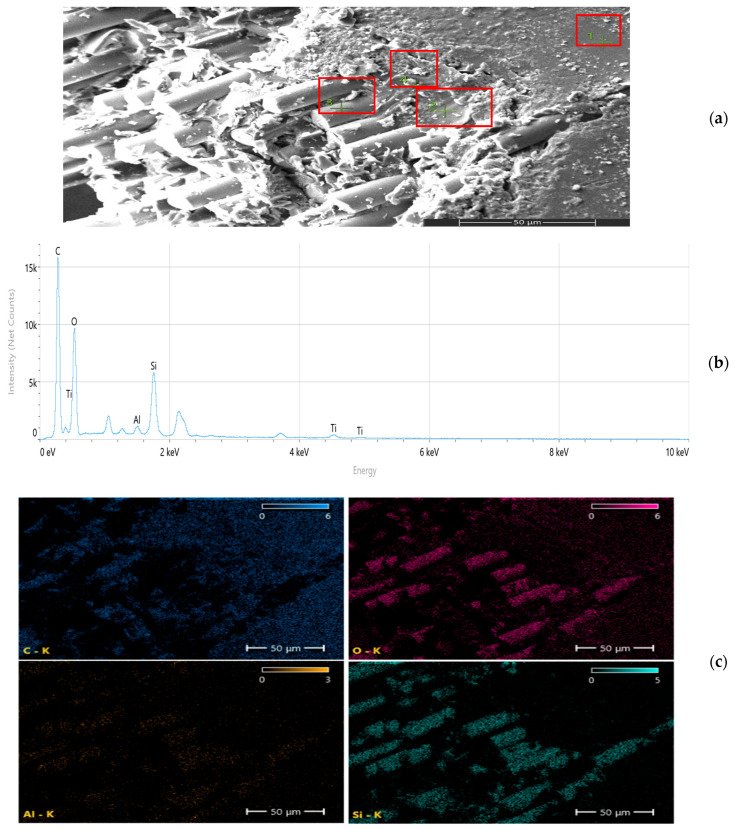
Microstructural and elemental characterization of the 8 wt% TiO_2_ composite: (**a**) SEM fracture morphology, (**b**) EDS spectrum, (**c**) elemental mapping.

At 8 wt% (Figure 25, Table 9), clear particle aggregation and matrix discontinuities are observed. Elemental mapping reveals intensified Ti concentration gradients, indicating localized modulus amplification zones and microcrack initiation regions.

Unlike Al_2_O_3_ systems, TiO_2_ composites exhibit smoother heterogeneity evolution, consistent with their more gradual FEM deviation trend.

Across all nanoparticle systems, spatial elemental gradients rather than nominal filler percentages govern the transition from dispersion-controlled strengthening to heterogeneity-driven stress amplification. These findings establish elemental mapping as direct microstructural evidence of the Composite Heterogeneity Threshold, providing a mechanistic bridge between morphology and continuum-level modeling deviation.

### 3.6. FEM Deviation and Continuum Model Validity Beyond the CHT

Continuum-based finite element models inherently rely on homogenized effective material properties and assume spatially uniform stiffness distributions. However, the combined SEM–EDS observations and SSDI analysis presented in Section 3.3, Section 3.4 and Section 3.5 demonstrate that beyond optimal nanoparticle loading, dispersion-induced heterogeneity generates localized stiffness gradients and interfacial stress concentration zones that cannot be fully represented within homogenized continuum formulations.

**Figure 26:**
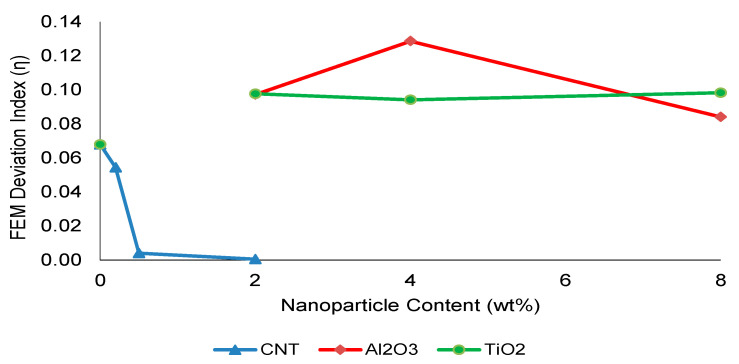
Variation in the FEM deviation index (η) with nanoparticle content, highlighting model sensitivity beyond the Composite Heterogeneity Threshold (CHT).

Figure 26 presents the evolution of the FEM deviation index (η) as a function of nanoparticle content. When interpreted together with the SSDI trends shown in Figure 8, a clear co-evolution beyond moderate filler loading becomes evident. In Al_2_O_3_-reinforced systems, η exhibits a pronounced peak at intermediate–high loading levels, coinciding with SSDI values exceeding unity. This behavior indicates the onset of stiffness-dominated heterogeneity, where additional modulus enhancement no longer translates into proportional strength improvement and numerical predictions increasingly deviate from experimental results.

In contrast, CNT-modified composites display comparatively stable and low η values across the investigated range. The minimal deviation observed at moderate CNT loading reflects a dispersion-controlled reinforcement regime in which homogenized orthotropic assumptions remain mechanically adequate. Similarly, TiO_2_-reinforced systems demonstrate relatively limited variation in η, consistent with their more gradual stiffness–strength evolution and progressive, rather than abrupt, heterogeneity development.

The concurrent increase in mechanical decoupling (SSDI) and numerical deviation (η), particularly in oxide-filled systems, confirms that the Composite Heterogeneity Threshold (CHT) represents not only a mechanical transition but also a model-validity boundary. Beyond this threshold, continuum-based representations progressively lose predictive reliability as microstructural instability and elastic mismatch dominate damage evolution.

Thus, the CHT framework establishes a direct linkage between dispersion stability, stiffness–strength decoupling, and continuum modeling adequacy, providing a unified criterion for assessing both mechanical efficiency and numerical reliability in nanoparticle-modified woven glass fiber composites.

## 4. Multi-Loading Mechanical Response and CHT Validation

**Figure 27:**
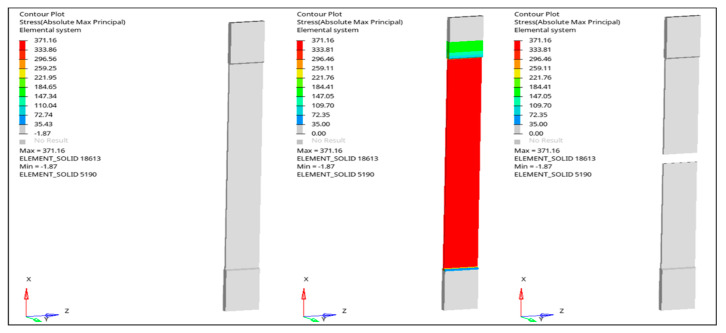
FEM-predicted maximum principal stress distribution in the neat GF composite under tensile loading.

**Figure 28:**
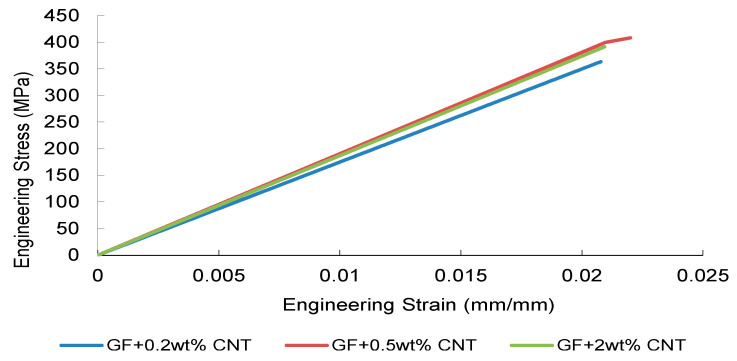
FEM-predicted engineering stress–strain curves of CNT-modified GF composites.

**Figure 29:**
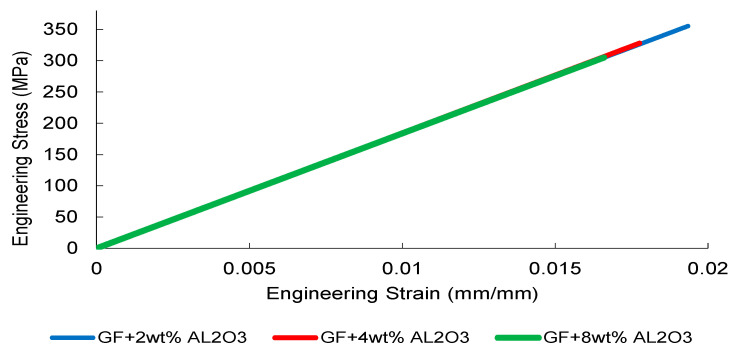
FEM-predicted engineering stress–strain curves of Al_2_O_3_-reinforced GF composites.

**Figure 30:**
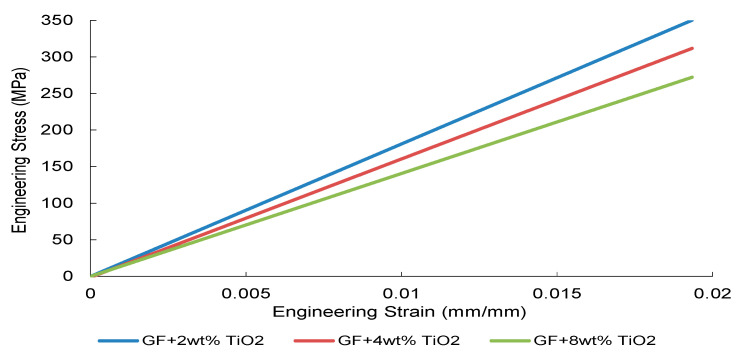
FEM-predicted engineering stress–strain curves of TiO_2_-reinforced GF composites.

The tensile simulations reproduced the experimentally observed linear-elastic response followed by peak stress and stiffness degradation, consistent with the strength-driven damage formulation implemented in the LS-DYNA MAT_022 framework (Figure 27, Figure 28, Figure 29 and Figure 30). The reference GF laminate exhibited uniform axial stress development within the gauge region (Figure 27), confirming numerical stability and appropriate displacement-controlled boundary conditions.

For CNT-modified systems (Figure 28), the simulations captured the non-monotonic reinforcement behavior, with the 0.5 wt% formulation showing minimal deviation from experimental peak strength. This response aligns with the dispersion-controlled regime identified in Section 3, where SSDI values remain close to unity and FEM deviation (η) is minimal.

In Al_2_O_3_-reinforced composites (Figure 29), stiffness enhancement at moderate loading was numerically reproduced; however, predicted peak stress reductions at elevated filler contents reflect the onset of stiffness-dominated heterogeneity. This trend corresponds directly to the Composite Heterogeneity Threshold (CHT) identified in Section 3.6, where concurrent increases in SSDI and η indicate the breakdown of homogenized continuum assumptions.

Similarly, TiO_2_-modified systems (Figure 30) exhibit progressive strength reduction with increasing filler content. The limited variation in SSDI and comparatively stable η values suggest gradual stress redistribution rather than abrupt heterogeneity-driven transitions, consistent with the microstructural interpretation presented earlier.

Overall, the tensile FEM results remain in agreement with the CHT framework, confirming that predictive accuracy deteriorates beyond the dispersion-controlled reinforcement window.

### 4.1. Flexural Response and Model Correlation

**Figure 31:**
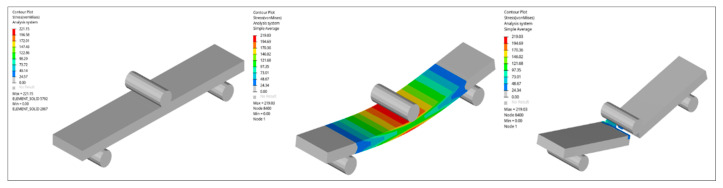
FEM contour plots showing the stress distribution in the neat GF composite during three-point bending.

**Figure 32:**
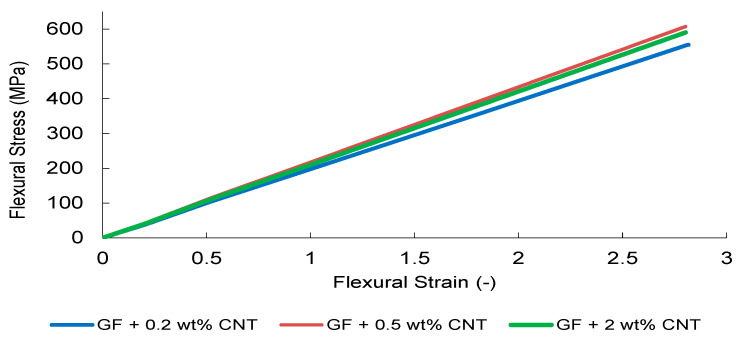
FEM-predicted flexural stress–strain curves of CNT-modified GF composites.

**Figure 33:**
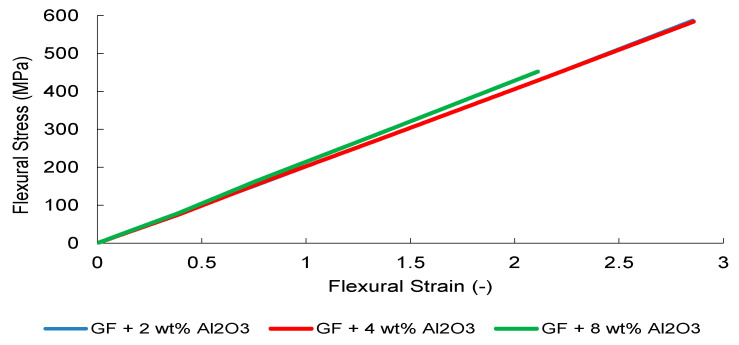
FEM-predicted flexural stress–strain curves of Al_2_O_3_-modified GF composites.

**Figure 34:**
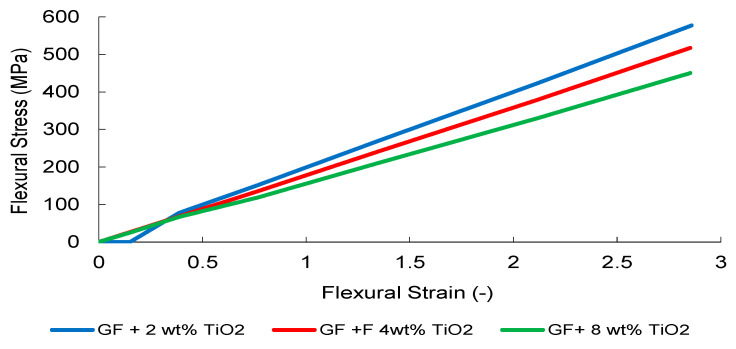
FEM-predicted flexural stress–strain curves of TiO_2_-modified GF composites.

**Figure 35:**
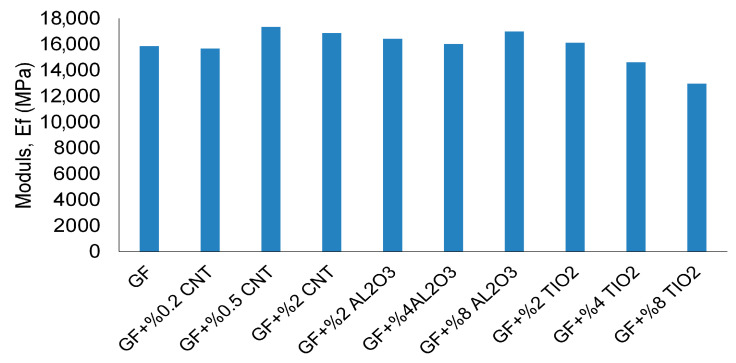
Comparison of flexural modulus (E_f) values derived from the initial linear region of FEM-predicted flexural stress–strain curves for CNT-, Al_2_O_3_-, and TiO_2_-reinforced GF composites.

Under three-point bending, all systems exhibited a quasi-linear load–deflection response up to peak load, followed by abrupt brittle fracture, consistent with tensile surface-controlled failure (Figure 31, Figure 32, Figure 33, Figure 34 and Figure 35). In contrast to uniaxial tension, bending introduces pronounced through-thickness strain gradients, localizing maximum tensile stresses at the outer surface. Consequently, flexural performance becomes highly sensitive to surface crack initiation, interfacial stability, and dispersion-induced stress redistribution.

CNT-modified composites demonstrate an optimal reinforcement window at 0.5 wt%, where the highest flexural stress response is observed (Figure 32). This behavior reflects effective crack-bridging within the tensile surface region and improved nanoscale stress transfer. However, increasing CNT content to 2 wt% resulted in strength reduction despite comparable initial stiffness, indicating dispersion-induced elastic heterogeneity and amplified strain gradients under bending. This sensitivity is more pronounced than in tensile loading, highlighting the influence of surface stress amplification beyond the Composite Heterogeneity Threshold (CHT).

Al_2_O_3_-reinforced systems exhibited progressive stiffness enhancement with increasing filler content (Figure 33). Although stiffness increases with filler content, the effective load-carrying capacity under bending is reduced at higher nanoparticle contents, indicating stiffness–strength decoupling. This stiffness–strength decoupling mirrors the transition identified in Section 3.6, where elevated particle loading promotes stiffness-dominated heterogeneity and localized interfacial stress concentration. Under bending, these effects intensify due to tensile surface stress localization, accelerating crack initiation despite increased bulk rigidity.

TiO_2_-modified composites (Figure 34) showed a monotonic reduction in effective flexural response despite slight increases in elastic slope, indicating limited strain accommodation and increased brittleness. Compared to CNT systems, no optimal reinforcement regime was observed, consistent with dispersion-controlled mechanisms governing load transfer.

The finite element simulations reproduce global stiffness trends with good qualitative agreement (Figure 31, Figure 32, Figure 33, Figure 34 and Figure 35). Stress contour distributions confirm classical bending behavior with tensile stress concentration at the lower surface and compressive stress at the upper surface (Figure 31), validating boundary conditions and mesh stability. However, predictive accuracy decreases at elevated filler contents, particularly in oxide systems, where homogenized continuum assumptions cannot fully resolve particle-scale agglomeration and interfacial debonding.

Overall, flexural loading amplifies microstructural heterogeneity effects relative to tensile loading, leading to earlier manifestation of the CHT. While global stiffness evolution remains reasonably captured, strength prediction deteriorates beyond the dispersion-controlled reinforcement window, indicating higher sensitivity of bending to localized stress amplification and strain gradients.

The larger deviations observed in flexural strength—particularly in the 4 wt% Al_2_O_3_ system—stem from the intrinsic through-thickness stress gradients and tensile surface localization inherent to bending-dominated loading. Because the MAT_022 formulation employs homogenized orthotropic strength parameters without explicitly resolving interlaminar damage or particle-scale clustering, localized stiffness amplification in oxide-rich systems may initiate experimental cracking earlier than predicted numerically.

Notably, the deviation escalation coincides with SSDI values exceeding unity, confirming that the discrepancy arises within the stiffness-dominated heterogeneity regime defined by the CHT. Therefore, the increased deviation represents a physically meaningful transition in damage sensitivity rather than a fundamental limitation of the continuum stiffness model. The co-evolution of SSDI and η under flexural loading further confirms that deviation escalation systematically aligns with the stiffness-dominated heterogeneity regime defined by the CHT, reinforcing the microstructure-governed nature of the transition.

### 4.2. Impact Response and Dynamic Validation of the CHT

**Figure 36:**
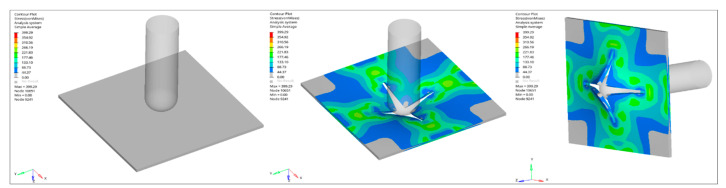
FEM-predicted stress distribution in the neat GF composite under low-velocity impact.

Low-velocity impact behavior was simulated using an explicit dynamic formulation in ANSYS LS-DYNA to resolve transient stress-wave propagation, contact interaction, and progressive damage evolution. The stress contour distributions of the reference GF laminate (Figure 36) show compressive stress localization beneath the impactor followed by radial redistribution toward the distal surface, consistent with bending-dominated impact behavior in thin laminates. The absence of artificial edge localization confirms numerical stability and appropriate time-step control.

**Figure 37:**
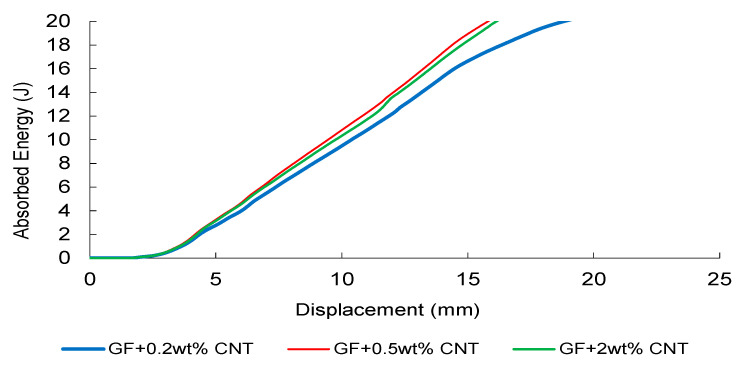
FEM-predicted energy–displacement curves of CNT-modified GF composites under low-velocity impact.

**Figure 38:**
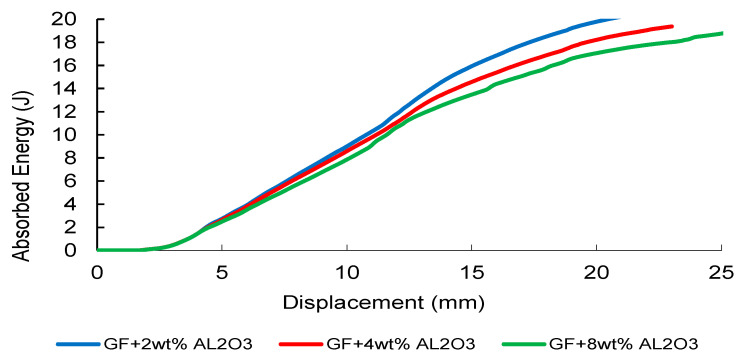
FEM-predicted energy–displacement curves of Al_2_O_3_-modified GF composites under low-velocity impact.

**Figure 39:**
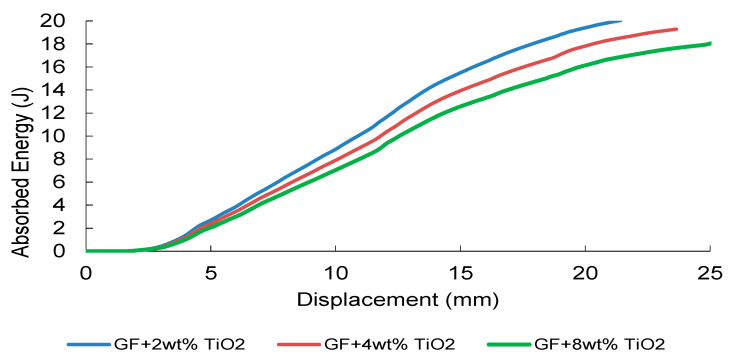
FEM-predicted energy–displacement curves of TiO_2_-modified GF composites under low-velocity impact.

The experimentally measured absorbed-energy–displacement responses exhibit a reinforcement-dependent evolution (Figure 37, Figure 38 and Figure 39). CNT-modified composites demonstrate a non-monotonic trend, with the 0.5 wt% CNT formulation achieving the highest total absorbed energy (Figure 37). This optimal window reflects enhanced stress-wave attenuation, effective crack-bridging, and delayed matrix-dominated damage initiation under transient loading. Increasing CNT content to 2 wt% leads to earlier energy saturation, indicating dispersion-induced elastic heterogeneity and localized damage activation. This transition represents the dynamic counterpart of the previously identified Composite Heterogeneity Threshold (CHT). Under dynamic loading, this transition is further amplified by stress-wave propagation effects, where impedance mismatch between matrix and nanoparticles leads to localized wave reflection and early damage initiation. This explains the reduced energy absorption efficiency at higher filler contents.

In addition, the observed energy absorption trends can be qualitatively associated with post-impact damage characteristics. Higher nanoparticle contents tend to promote localized damage zones, including matrix cracking and interfacial debonding, due to increased stiffness mismatch. In contrast, optimally dispersed systems exhibit more distributed damage patterns, indicating enhanced energy dissipation through progressive damage mechanisms.

Al_2_O_3_-reinforced systems (Figure 38) show comparable energy absorption at moderate filler levels (2–4 wt%), suggesting stable load redistribution during initial indentation. However, at 8 wt%, total absorbed energy decreases noticeably, accompanied by earlier plateau formation. This behavior reflects stiffness-dominated reinforcement, where increased modulus contrast accelerates interfacial stress concentration and matrix cracking under impact conditions.

TiO_2_-modified composites (Figure 39) display a systematic reduction in absorbed energy with increasing filler fraction. Although moderate additions slightly enhance initial contact stiffness, sustained energy dissipation capacity decreases progressively. The dominant modulus mismatch between rigid TiO_2_ particles and the polymer matrix promotes stress-wave reflection and localized tensile damage initiation at the distal surface.

**Figure 40:**
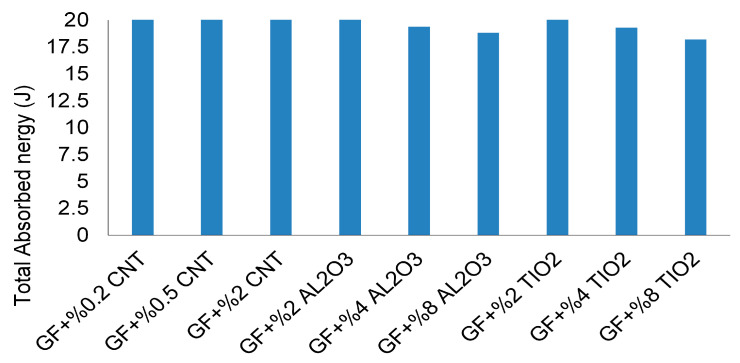
Total absorbed energy of CNT-, Al_2_O_3_-, and TiO_2_-modified GF composites under low-velocity impact.

The comparative summary of total absorbed energies (Figure 40) confirms a non-proportional relationship between filler content and dynamic performance. While CNT systems exhibit a clear optimal reinforcement window, oxide-filled systems demonstrate stiffness-controlled impact behavior with limited contribution to progressive damage stabilization. Experimental–numerical comparison (Table 10) indicates acceptable deviation levels overall; however, larger discrepancies in CNT and TiO_2_ systems at elevated filler contents highlight the limitations of homogenized continuum assumptions under highly transient, localized damage conditions.

Collectively, the impact results validate the existence of a critical heterogeneity threshold beyond which elastic mismatch and localized stress amplification dominate dynamic damage evolution. The consistency between tensile, flexural, and impact transitions confirms that the CHT framework governs mechanical response across static and dynamic loading regimes.

The large deviations observed in tensile strength are primarily attributed to the homogenized nature of the FEM model, which does not explicitly capture nanoparticle agglomeration, interfacial defects, and local stress concentrations. These effects become particularly critical under tensile loading, where failure is highly sensitive to microscale damage initiation and dispersion instability.

It is important to emphasize that the FEM model represents an idealized, perfectly dispersed composite with uniform interfacial bonding, whereas the experimental system inherently contains heterogeneities such as particle clustering and imperfect interfaces. As a result, the FEM predictions tend to overestimate the load-carrying capacity, especially at higher nanoparticle contents.

Therefore, the observed deviations should not be interpreted as numerical inconsistencies, but rather as physically meaningful indicators of microstructural heterogeneity. Within the proposed Composite Heterogeneity Threshold (CHT) framework, the increase in FEM deviation directly reflects the transition from dispersion-controlled reinforcement to heterogeneity-dominated mechanical behavior. Across tensile, flexural, and impact loading conditions, a consistent reinforcement-dependent transition is observed. While tensile loading reveals the initial manifestation of the Composite Heterogeneity Threshold (CHT), flexural loading amplifies this transition due to surface tensile stress localization and strain-gradient effects. Under impact conditions, transient stress-wave propagation further intensifies elastic mismatch and localized damage initiation, representing the dynamic counterpart of the same heterogeneity-controlled mechanism. This cross-loading consistency demonstrates that the CHT is not loading-specific but represents a fundamental microstructure-governed transition, directly linking dispersion stability to predictive reliability and mechanical performance across static and dynamic regimes.

## 5. Conclusions

This study systematically investigated the mechanical behavior of CNT-, Al_2_O_3_-, and TiO_2_-modified woven glass fiber composites through combined experimental and numerical approaches. The main findings are summarized as follows:

- Mechanical performance is primarily governed by nanoparticle dispersion stability rather than nominal filler content.
- CNT-modified composites exhibit an optimal reinforcement regime at 0.5 wt%, where simultaneous improvements in tensile, flexural, and impact properties are achieved. Beyond this level, agglomeration leads to interfacial degradation and reduced mechanical performance.
- Oxide nanoparticle systems (Al_2_O_3_ and TiO_2_) demonstrate stiffness-dominated reinforcement behavior, where increasing filler content enhances modulus but induces elastic mismatch, particle clustering, and localized stress concentration.
- The proposed Strength–Stiffness Decoupling Index (SSDI) and FEM deviation index (η) enable quantitative identification of a Composite Heterogeneity Threshold (CHT), marking the transition from dispersion-controlled to heterogeneity-dominated behavior.
- Flexural loading is particularly sensitive to heterogeneity due to surface tensile stress localization and strain-gradient effects, while impact loading reflects the dynamic manifestation of the same transition through stress-wave-driven damage initiation.
- The consistent emergence of the CHT across tensile, flexural, and impact loading conditions confirms that it represents a microstructure-governed material state boundary rather than a loading-specific phenomenon.
- While the homogenized orthotropic FEM model accurately predicts global stiffness within the dispersion-controlled regime, its predictive capability decreases beyond the CHT due to particle clustering and interfacial instability.

Overall, this study establishes a unified mechanistic framework linking nanoparticle dispersion, stiffness–strength decoupling, and continuum model validity. The CHT concept provides a robust design and modeling criterion for optimizing nanoparticle-reinforced composite systems under complex loading conditions.

## 6. Future Outlook

The findings of this study open several directions for future research and practical applications. The concept of the Composite Heterogeneity Threshold (CHT) can be further extended to hybrid and multi-scale reinforcement systems, where multiple nanoparticle types are combined to tailor both stiffness and toughness simultaneously.

Future studies should focus on developing microstructure-resolved modeling approaches that explicitly account for particle clustering, interfacial damage, and local stress gradients beyond the CHT. Such approaches would improve the predictive capability of numerical models under high filler content conditions.

From an application perspective, the identification of optimal dispersion regimes provides valuable guidance for designing high-performance composite structures in aerospace, automotive, and impact-resistant systems, where multi-axial loading conditions are critical.

In addition, advanced processing techniques aimed at improving nanoparticle dispersion at higher loadings could enable the extension of the reinforcement window while delaying the onset of heterogeneity-driven degradation.

## Figures and Tables

**Table 1:** Matrix formulations and corresponding nanoparticle loadings of the fabricated composite laminates.

Nanoparticle—Free GF Composite (0 wt%)	Epoxy Resin (gr)	Epoxy Hardener (gr)	Nanoparticle (gr)
	200	100	-
%0.2 CNTs	199.6	99.8	0.6
%0.5 CNTs	199	99.5	1.5
%2 CNTs	196	98	6
%2 Al_2_O_3_	196	98	6
%4 Al_2_O_3_	192	96	12
%8 Al_2_O_3_	184	92	24
%2 TiO_2_	196	98	6
%4 TiO_2_	192	96	12
%8 TiO_2_	184	92	24

**Table 2:** Orthotropic elastic properties used in FEM simulations derived from experimental measurements.

Property	GF	0.2 wt% CNTs	0.5 wt% CNTs	2 wt% CNTs	2 wt% Al_2_O_3_	4 wt% Al_2_O_3_	8 wt% Al_2_O_3_	2 wt% TiO_2_	4 wt% TiO_2_	8 wt% TiO_2_
Eₓ (MPa)	17,582	17,485	19,050	18,700	18,369.67	18,461.10	19,691.84	18,100.67	16,175.44	14,065.60
Eᵧ (MPa)	17,582	17,485	19,050	18,700	18,369.67	18,461.10	19,691.84	18,100.67	16,175.44	14,065.60
E_z_ (MPa)	17,582	17,485	19,050	18,700	18,369.67	18,461.10	19,691.84	18,100.67	16,175.44	14,065.60
ν_xy_	0.072	0.088	0.10	0.086	0.075	0.068	0.090	0.074	0.080	0.075
νₓ_z_	0.072	0.088	0.10	0.086	0.075	0.068	0.090	0.074	0.080	0.075
ν_yz_	0.072	0.088	0.10	0.086	0.075	0.068	0.090	0.074	0.080	0.075
G_xy_ (MPa)	1125	1515	2000	1522	1175.40	1068.75	990	1158.19	1035	900
Gₓ_z_ (MPa)	1125	1515	2000	1522	1175.40	1068.75	990	1158.19	1035	900
G_yz_ (MPa)	1125	1515	2000	1522	1175.40	1068.75	990	1158.19	1035	900

Note: E_x_, E_y_, and E_z_ denote the elastic moduli along the principal material directions; ν_xy_, ν_xz_, and ν_yz_ represent the corresponding Poisson’s ratios; G_xy_, G_xz_, and G_yz_ denote the shear moduli. These orthotropic properties were derived based on experimentally obtained tensile data and used as input parameters in the FEM simulations.

**Table 3:** GF Composite Atomic Contents.

Element	At.%	wt%	Net Counts
C	58.4	47.2	51,303
O	31.5	33.9	53,884
Al	0.7	1.2	5195
Si	9.4	17.7	73,678
Ti	0.0	0.0	37

**Table 4:** 0.5 wt% CNT Composite Atomic Contents.

Element	At.%	wt%	Net Counts
C	58.6	47.2	104,077
O	31.1	33.3	108,971
Al	0.6	1.1	10,272
Si	9.7	18.3	158,201
Ti	0.0	0.1	189

**Table 5:** 2 wt% CNT Composite Atomic Contents.

Element	At.%	wt%	Net Counts
C	59.3	47.8	218,561
O	30.1	32.3	217,202
Al	0.7	1.2	23,060
Si	9.9	18.6	331,105
Ti	0.0	0.1	275

**Table 6:** 2 wt% Al_2_O_3_ Composite Atomic Contents.

Element	At.%	wt%	Net Counts
C	60.1	49.5	33,772
O	30.3	32.7	55,749
Al	0.8	1.3	6050
Si	8.8	16.5	92,258
Ti	0.0	0.1	244

**Table 7:** 8 wt% Al_2_O_3_ Composite Atomic Contents.

Element	At.%	wt%	Net Counts
C	56.6	45.8	106,041
O	34.1	36.7	123,747
Al	1.6	2.9	27,102
Si	7.7	14.6	129,475
Ti	0.0	0.0	9

**Table 8:** 2 wt% TiO_2_ Composite Atomic Contents.

Element	At.%	wt%	Net Counts
C	78.7	70.5	174,552
O	19.0	22.8	32,296
Al	0.0	0	0
Si	1.1	2.4	11,170
Ti	1.2	4.3	3962

**Table 9:** 8 wt% TiO_2_ Composite Atomic Contents.

Element	At.%	wt%	Net Counts
C	64.9	54.2	88,266
O	28.3	31.3	52,103
Al	0.4	0.8	4043
Si	5.4	10.4	52,513
Ti	1.0	3.3	3299

**Table 10:** Comparison of Mechanical Properties between Experimental Results and FEM Predictions (trend-based evaluation).

Material	Tensile Strength (MPa)			Flexural Strength (MPa)			Impact Test (J)		
	Test	FEM	Difference (%)	Test	FEM	Difference (%)	Test	FEM	Difference (%)
GF	169.31	371.16	−119.23	182.55	219.03	−19.98	9.53	8.55	10.29
%0.2 CNTs	138.62	392.7	−183.38	194.11	216.68	−11.63	8.55	8.42	1.54
%0.5 CNTs	153.21	417.77	−172.66	230.23	237.66	−3.23	9.08	8.51	6.24
%2 CNTs	117.83	408	−246.28	195.56	231.21	−18.23	9.64	8.56	11.23
%2 Al_2_O_3_	166.62	397.82	−138.77	190.15	228.8	−20.32	9.05	8.42	6.90
%4 Al_2_O_3_	151.11	372.03	−146.19	172.90	228.75	−32.30	7.87	8.07	−2.49
%8 Al_2_O_3_	154.01	331.04	−114.96	160.16	176.26	−10.05	7.28	7.84	−7.68
%2 TiO_2_	147.28	392.14	−166.27	187.37	225.36	−20.28	8.91	8.35	6.25
%4 TiO_2_	176.45	349.28	−97.97	167.44	201.53	−20.36	7.35	8.04	−9.42
%8 TiO_2_	169.72	304.89	−79.63	145.60	175.3	−20.40	6.72	7.58	−12.81

## Data Availability

The original contributions presented in this study are included in the article. Further inquiries can be directed to the corresponding author.
